# DeephESC 2.0: Deep Generative Multi Adversarial Networks for improving the classification of hESC

**DOI:** 10.1371/journal.pone.0212849

**Published:** 2019-03-06

**Authors:** Rajkumar Theagarajan, Bir Bhanu

**Affiliations:** 1 Depratment of Electrical and Computer engineering, University of California, Riverside, Riverside, CA, United States of America; 2 Center for Research in Intelligent Systems, University of California, Riverside, Riverside, CA, United States of America; 3 Department of Bioengineering, University of California Riverside, Riverside, CA, United States of America; Universidad de Granada, SPAIN

## Abstract

Human embryonic stem cells (hESC), derived from the blastocysts, provide unique cellular models for numerous potential applications. They have great promise in the treatment of diseases such as Parkinson’s, Huntington’s, diabetes mellitus, etc. hESC are a reliable developmental model for early embryonic growth because of their ability to divide indefinitely (pluripotency), and differentiate, or functionally change, into any adult cell type. Their adaptation to toxicological studies is particularly attractive as pluripotent stem cells can be used to model various stages of prenatal development. Automated detection and classification of human embryonic stem cell in videos is of great interest among biologists for quantified analysis of various states of hESC in experimental work. Currently video annotation is done by hand, a process which is very time consuming and exhaustive. To solve this problem, this paper introduces DeephESC 2.0 an automated machine learning approach consisting of two parts: (a) Generative Multi Adversarial Networks (GMAN) for generating synthetic images of hESC, (b) a hierarchical classification system consisting of Convolution Neural Networks (CNN) and Triplet CNNs to classify phase contrast hESC images into six different classes namely: *Cell clusters*, *Debris*, *Unattached cells*, *Attached cells*, *Dynamically Blebbing cells* and *Apoptically Blebbing cells*. The approach is totally non-invasive and does not require any chemical or staining of hESC. DeephESC 2.0 is able to classify hESC images with an accuracy of 93.23% out performing state-of-the-art approaches by at least 20%. Furthermore, DeephESC 2.0 is able to generate large number of synthetic images which can be used for augmenting the dataset. Experimental results show that training DeephESC 2.0 exclusively on a large amount of synthetic images helps to improve the performance of the classifier on original images from 93.23% to 94.46%. This paper also evaluates the quality of the generated synthetic images using the Structural SIMilarity (SSIM) index, Peak Signal to Noise ratio (PSNR) and statistical *p*-value metrics and compares them with state-of-the-art approaches for generating synthetic images. DeephESC 2.0 saves hundreds of hours of manual labor which would otherwise be spent on manually/semi-manually annotating more and more videos.

## 1 Introduction and background

Human embryonic stem cells (hESC) are derived from the inner cell mass of developing blastocysts and can be maintained indefinitely in vitro in a pluripotent state [1]. hESC have the ability to self-renew and differentiate into any cell type, thus providing a unique resource for regenerative medicine and toxicological testing of drugs [2, 3]. The biologists who study hESC have to manually analyze stem cell videos every day. On an average it takes 3-5 days for a biologist to manually analyze a single hESC video, taken over a period of 48 hours with a suitable sampling rate, and annotate its different stages of development. To date, there are very limited automated tools [4, 5] for classifying hESC from videos making it a very laborious manual process.

**Fig 1:**
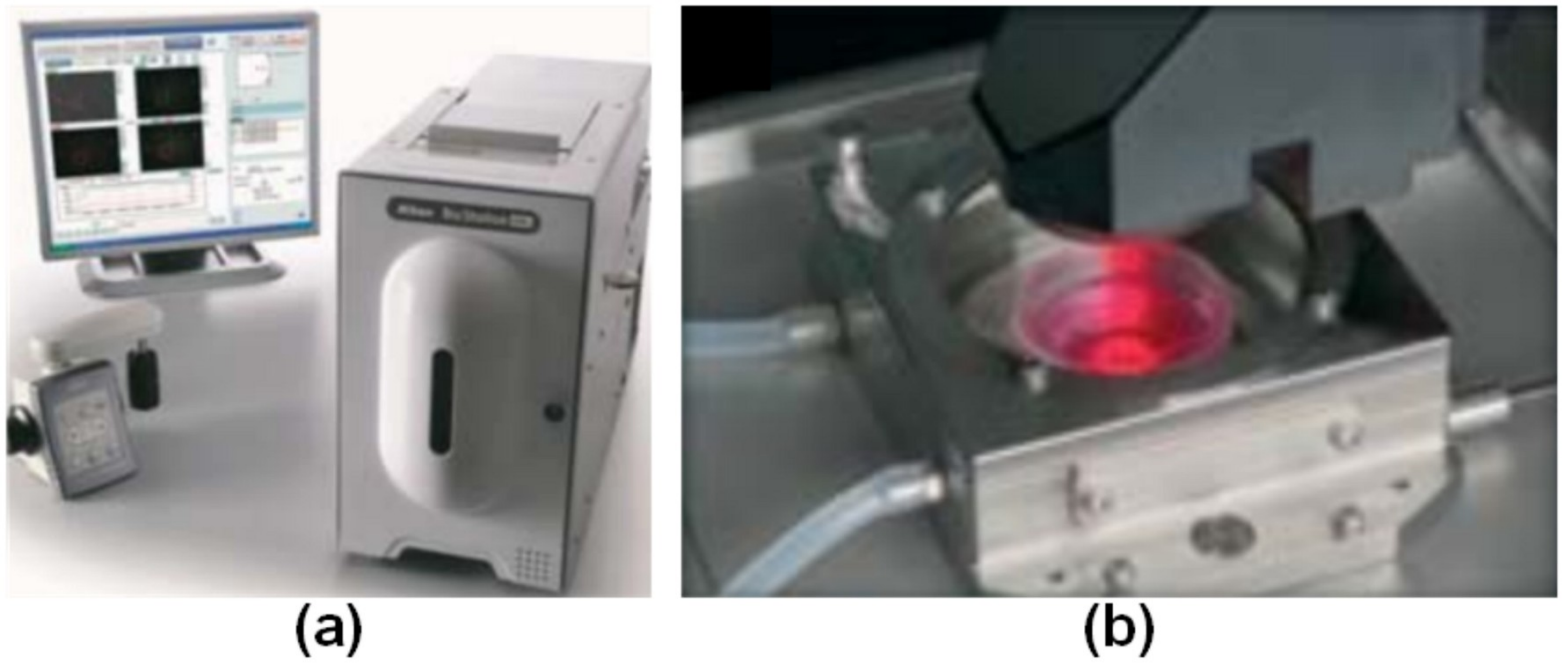
The Nikon BioStation IM benchtop live cell imaging system. (a) External features include a incubation unit, joystick for controlling the position of the camera during sample selection, and a monitor. (b) Culture dish sitting inside the BioStation IM incubator unit.

**Fig 2:**
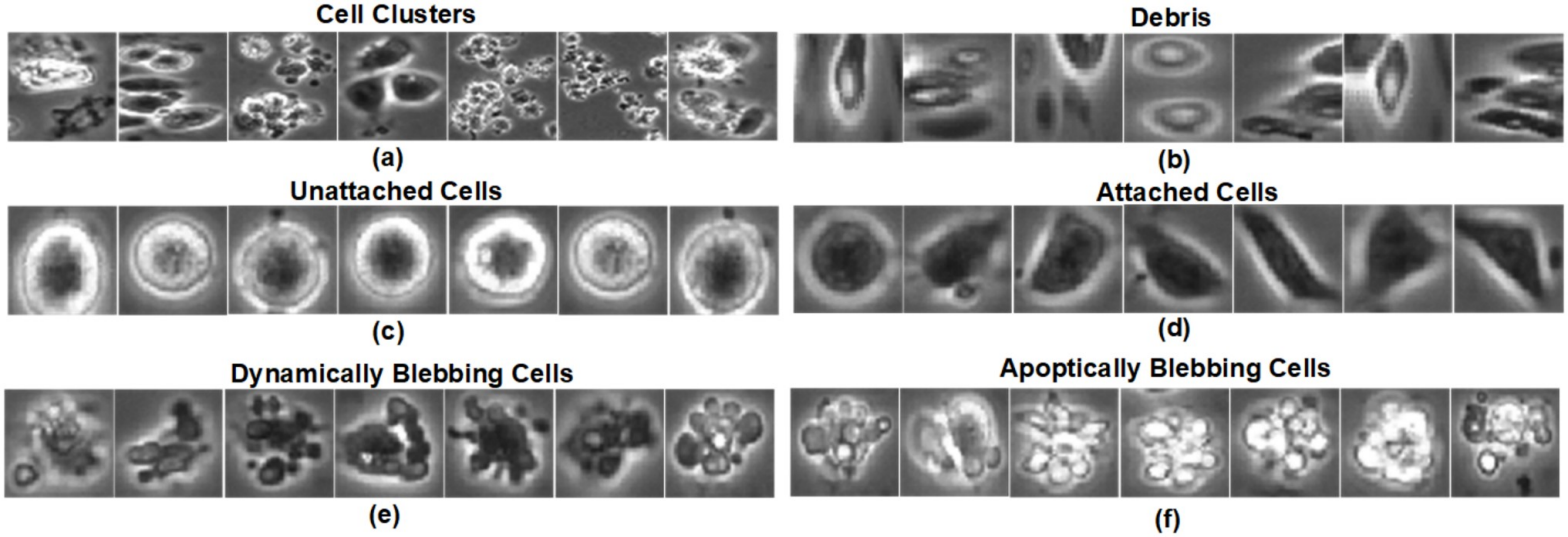
Phase contrast images for the six different classes of hESC obtained from the Nikon BioStation IM.

Video Bioinformatics [6]–[10] is an upcoming field to help biologists use efficient and effective approaches to analyze expansive volumes of video data. In this study, the hESC videos were recorded using a Nikon BioStation IM [11] which has a phase contrast microscope. Each frame in the video can contain any number of the following six cell types: 1) *Cell clusters **(CC)***, 2) *Debris* ***(DEB)***, 3) *Unattached Cells **(UN)***, 4) *Attached Cells **(AT)***, 5) *Dynamically Blebbing Cells **(DYN)***, and 6) *Apoptotically Blebbing cells **(APO)***. Fig 1 shows the Nikon BioStation IM and Fig 2 shows the hESC phase contrast images that have been detected and cropped from full frame images for each class. **It should be noted that, our approach is totally non-invasive and does not require chemicals for staining the hESC.**

The *Unattached cells*, *Attached cells*, *Dynamically Blebbing cells* and *Apoptically Blebbing cells* are considered as the intrinsic cell types. *Cell clusters* are a colony of growing cells consisting of a group of two or more different intrinsic cell types that are packed close to each other. Blebbing cells are membrane protrusions that appear and disappear from the surface of cells. The changing area of the blebbing cells over time is important for understanding and evaluating the health of cells. *Dynamic blebs* indicate healthy cells and *Apoptotic blebs* indicate dying cells. The ability to analyze rates of bleb formation and retraction are important in the field of toxicology and could form the basis of an assay that depends on a functional cytoskeleton [12].

From Fig 2, it can be observed that although certain classes such as *Debris* and *Unattached cells* look very discriminative compared to the remaining four classes. Certain classes like *Attached cells* and *Dynamically Blebbing cells* share very similar color intensities, similarly *Cell clusters* and *Apoptically Blebbing cells* share very similar texture making making it very challenging to classify these hESC classes.

Previous studies involving the classification of hESC have primarily used manual/ semi-manual detection and segmentation [13], hand-crafted feature extraction [4]. These manual methods, hand-crafted feature extraction approaches are prone to human bias and they are tedious and time-consuming processes when performed on a large volume of data. Therefore, it is advantageous to develop an image analysis software such as DeephESC 2.0 to automatically classify hESC images and also generate synthetic data to compensate for the lack of real data.

Recent years have witnessed the boom of CNNs in many computer vision and pattern recognition applications including object classification [14], object detection [15] and semantic segmentation [16]. In this paper, we propose DeephESC 2.0, an automated machine learning based classification system for classifying hESC images using Convolution Neural Networks (CNN) and Triplet CNNs in a hierarchical system. The CNNs are trained on a very limited dataset consisting of phase contrast imagery of hESC to extract discriminative and robust features to automatically classify these images. This is not a straight forward task as some classes of hESC have very similar shape, intensity and texture. To solve this we trained triplet CNNs that help extract very fine-grained features and classify between two very similar but slightly distinctive classes of hESC. DeephESC 2.0 uses a CNN and two triplet CNNs fused together in a hierarchical manner to perform fine-grained classification on six different classes of hESC images. Previous studies have shown that augmenting the size and diversity of the dataset, results in improved classification accuracy [17].

The process of obtaining video recordings of hESC is a very long and tedious process, and to date there are no publicly available datasets. To compensate for the lack of data, DeephESC 2.0 uses Generative Multi Adversarial Networks (GMANs) to generate synthetic hESC images and augment the training dataset to further improve the classification accuracy. We compare different architectures of Generative Adversarial Networks (GANs) and the quality of the generated synthetic images using the Structural SIMilarity (SSIM) index and Peak Signal to Noise Ratio (PSNR). Furthermore, we trained DeephESC 2.0 using the synthetic images, evaluated it on the original hESC images obtained from biologists and verified the significance of our results using the *p*-value.

This paper is organized as follows. Section 2 describes the related work and our contributions for detecting, classifying and generating synthetic hESC images. The data and technical approach are presented in Section 3. Experimental results are discussed in detail in Section 4, followed by the conclusions of our paper given in Section 5.

## 2 Related work and our contributions

Some preliminary work reported in this paper was originally presented at the International Conference on Pattern Recognition 2018 [5]. To the best of our knowledge, before our previous conference paper [5], hESC images have never been automatically classified into six different classes using synthetically generated image data. In the following we present the related work into the following three areas: detection of hESC in video, classification of hESC images and generation of synthetic hESC images.

### 2.1 Detection of hESC in video

There are some current methods for detecting cell regions in phase contrast images [4]. Table 1 shows the summary of the related work done for detecting hESC. Ambriz-Colin *et al*. [18] proposed two methods for cell region detection from phase contrast images: detection by pixel Intensity Variance (PIV) and detection by Gray Level Morphological Gradient (GLMG). The PIV method computes the variance of a pixel in a given neighborhood and based on a threshold classifies if the pixel belongs to a cell region or background. The GLMG approach converts the phase contrast image to a binary image and performs morphological dilation and erosion and based on a threshold separates the cell region and background. Li *et al*. [19] used a combination of morphological rolling-ball filtering and a Bayesian classifier to classify the pixels into either the cell regions or the background. The major drawback with these approaches is that they are very susceptible and would fail to classify the pixels even if there is slight change in pixel intensity or change in texture which normally occurs over time.

**Table 1:** Summary of the related work for detecting hESC.

**Detection of hESC**	**Authors**	**Comments**
	Ambriz-Colin *et al*. [18]	Used the Pixel Intensity Variance (PIV) and Gray Level Morphological Gradient (GLMG) for detecting cell regions from phase contrast images.
	Li *et al*. [19]	Used a combination of morphological rolling-ball filter and a Bayesian classifier to classify pixels into cell regions and background.
	Eom *et al*. [20]	Used circular Hough transform to detect the shapes of cells in an image. This approach is sensitive to variations in the shape.
	Miroslaw *et al*. [21]	Used template based correlation to detect cell bodies. This approach requires pre-selecting exemplary cell body images as a template.
	Tatiraju *et al*. [22]	Used a variant of the K-means algorithm to segment the cell bodies. This approach fails in separating cell clusters that are close to each other.
	Zarpak *et al*. [23]	Used multiple Gaussian models to represent the pixel intensity distribution of the cell body. This approach ignores the connectivity between adjacent cell clusters.
	**Guan** ***et al***. [24]	**Used individual mixture of Gaussian models to model the pixel intensity of the foreground cell body and background substrate. This approach also uses the local spatial information of cell bodies to separate adjacent clusters close to each other.**

Eom *et al*. [20] used circular Hough transform to detect the shapes of cells in an image and classify them. This approach is very sensitive to the variation of shapes and appearance of cells. This approach is not viable for detection of hESC where blebbing is continuously altering the shape of the hESC. Miroslaw *et al*. [21] proposed to use correlation using template images for cell region detection. This approach requires pre-selection of exemplar template images which are not readily available in most cases. Moreover, this approach is most likely to fail in conditions where parts of two or more cells are overlapping in a single image.

The most commonly used algorithms for image segmentation are the K-means segmentation and mixture of Gaussians by Expectation-Maximization (EM) algorithm. Tatiraju *et al*. [22] used a variant of the K-means algorithm such that each pixel intensity is considered as an individual observation and the authors partition these observations into *k* clusters. This method does not consider the intensity distribution of its clusters. As a result the segmentation obtained lacks the connectivity within the neighborhood pixels. The mixture of Gaussians segmentation proposed by Farnoosh and Zarpak [23] depends heavily on the intensity distribution models to group the image data. The underlying assumption of their approach is that intensity distribution of the image can be represented by multiple Gaussians. However, it does not take into account the neighborhood information. As a result, the segmented regions lack connectivity with the pixels within their neighborhood.

DeephESC 2.0 detects the hESC regions using the approach proposed by Guan *et al*. [24]. The algorithm uses the intensity distributions of the foreground (hESC) and background (substrate) as well as the cell property for detection. The intensity distributions of the foreground and background are modeled as a mixture of two Gaussians and the cell property is translated into a local spatial information. The algorithm is optimized by parameters of the distributions and the cell regions evolve with the local cell property. The advantage of this approach is that, it not only uses information of the foreground and background, but it also uses cell properties resulting in fine-grained localization of the hESC even in the presence of background noise.

### 2.2 Classification of hESC images

Although there has been some work for detecting cell regions from phase contrast images, there is very limited work done for classifying them into different classes. Table 2 shows the summary of the related work done for classifying hESC. Lowry *et al*. [25] designed a texture based multi-stage Bayesian level set algorithm to segment pluripotent and trophectoderm colony images of hESC and their derivatives. The authors used an MR8 approach [26] for modeling the texture by convolving image patches with a filter bank containing Gaussian and Laplacian of Gaussian (LoG) filters at a fixed scale and edge bar filters at three different scales and several orientations. This results in a texton feature vector containing eight filter responses for every given pixel in the image. After extracting these texton features, the texturally inhomogeneous images are segmented using a multi stage Bayesian Level Set (BLS). The advantage of using BLS is that it produces smoother segmentation maps with regular borders and is much more tolerant to poor initial conditions.

**Table 2:** Summary of the related work for classification of hESC and contributions of this paper.

**Classification of hESC**	Lowry *et al*. [25]	Used Gaussians and Laplacian of Gaussian (LoG) filters to obtain a texton feature vector for each pixels and then use a multi-stage Bayesian set algorithm to segment and classify pluripotent and trophectoderm colonies.
	Lowry *et al*. [27]	Used an adjustable length window to extract features using multi-resolution wavelet analysis which is used to classify the cell nuclei using a combination of set levels and non-parametric estimation of the wavelet coefficients.
	Mangoubi *et al*. [28]	Used a wavelet texture decomposition and a set of visual features to distinguish between pluripotent and differentiated colonies. Based on the results, the authors suggest that a good pluripotent colony must exhibit a tight homogeneous texture.
	Desai *et al*. [29]	Used a matrix edge function to classify stem cell nucleus into pluripotent and differentiating nucleus. This approach assumes that the nucleus exhibits an onion layer texture where the texture is homogeneous within the layer and varies between different layers.
	Sammak *et al*. [30]	Used a wavelet decomposition and matrix edge function to extract features which are given to Support Vector Machine (SVM) to classify differentiating cells into Trophectoderm, Neurectoderm, and Progeny cells.
	Niioka *et al*. [31]	Used CNNs and the morphological changes of the differentiating cell body to detect and classify the differentiation from myoblasts to myotubes.
	Chang *et al*. [32]	Classified 256×256 image patches of human Induced Pluripotent Stem cells in human cord blood CD34++ images using Convolutional Neural Networks (CNNs).
	Xie *et al*. [33]	Used a CNN to localize and count the number of individual cells in fluorescent images.
	Witmer *et al*. [34]	Used entropy based filters, CNNs and patches extracted from a sliding 224x224 window to segment and classify cell colonies into six different sub-colonies.
	Theagarajan *et al*. [5]	Used a CNNs to classify hESC images into six different classes. Although this approach achieves high classification accuracy, it has a high error rate in classifying classes that visually have a similar texture such as Cell clusters/Apoptically Blebbing cells and Attached/Dynamically Blebbing cells.
	**Our Approach**	**This paper uses a combination of CNN and triplet CNNs in a hierarchical system to classify phase contrast hESC images into six different classes. This non-invasive approach uses skip connections between convolutional layers which helps to learn more robust features and also improves the classification between classes that have very similar textures.**

Lowry *et al*. [27] combined set levels, multi resolution wavelet analysis and non- parametric estimation of the density functions of the wavelet coefficients to segment and classify stem cell nuclei. The authors also used an adjustable length window to deal with small size textures where the largest inscribed rectangular window may not contain a sufficient number of pixels for multiresolution analysis of elongated and irregularly shaped nuclei. Mangoubi *et al*. [28] classified hESC into differentiated and pluripotent cell colonies using a wavelet based texture decomposition. The authors used four visual features namely: *textural homegeneity*, *textural tightness*, *border sharpness* and *border circularity*. Based on these visual features, the authors achieved an accuracy of 96% in classifying colonies that were very distinct from each other and 86% in colonies with a mixed distribution. The authors suggest that a good pluripotent stem cell colony must exhibit a homogeneous, tight texture throughout, thus allowing a statistical analysis of the coefficients obtained from a wavelet based texture decomposition to discriminate between the colonies.

Desai *et al*. [29] classified fluorescent stem cell nucleus images into pluripotent and differentiated nucleus. Stem cell nuclei are very small in size and have very few pixels on them. The authors assume that the nucleus exhibits an onion layer texture where we may assume that within a layer the behavior is homogeneous, but may vary from layer to layer. The authors use a matrix edge function that adaptively modulates the shape, size, and orientation of neighborhoods over different regions of the texture, thus providing directional information on the texture that is not available in the more conventional scalar edge field based approaches.

Sammak *et al*. [30] classified differentiating cells into three classes namely: *Trophectoderm, Neurectoderm, and Progeny cells*. The authors showed that during differentiation the edges at the borders of the cell become more thin. The authors extract features, using wavelet decomposition and a matrix edge function, which are then given to a Support Vector Machine (SVM) for classification.

Niioka *et al*. [31] detect the cellular differentiation of myoblasts to myotubes using Convolutional Neural Networks. During the differentiation process, the cellular morphology changes from a round shape to an elongated tubular shape due to the fusion of cells. The authors trained their CNN using stained fluorescent images as input and were able to detect the differentiation with an accuracy of 91.3%.

Chang *et al*. [32] were able to classify human Induced Pluripotent Stem (iPS) cells in human cord blood CD34+ images using Convolutional Neural Networks. The authors used a 5 convolutional layer network to classify 256x256 patches of images with an accuracy of 91.8%. Xie *et al*. [33] performed cell counting in fluorescent images using a convolutional regression network. They trained a network to localize fluorescent labeled cell nuclei via down-convolutional feature extraction and symmetrically up-convolutional pixel-wise classification. They apply their network to a variety of datasets and manually annotated grayscale histology sections with an average error of 2.9% for their cell counting task. While their method is a successful implementation for training a neural network feature classifier for localizing cells, it is relatively easy to localize cells in a fluorescent dataset compared to more non-invasive complex datasets, such as the data with low contrast and high texture. A drawback of the works done by [31], [32] and [33] is that they had to stain hESC in order to classify them making it an invasive approach, whereas, our approach is totally non-invasive.

Witmer *et al*. [34] developed an automated system to localize six cell colonies namely: *Debris*, *Dense*, *Spread*, *Differentiated*, *Partially spread*, and *Partially differentiated*. The authors extracted patches of size 224 × 224 using a sliding window from phase contrast images. These patches are then passed through an entropy filter that segments the cell colonies by exploiting the difference between the background and foreground of the images. The segmented patches are then passed through a CNN which classifies the patch into one of the 6 classes. The authors were able to achieve an accuracy of 89.35% in classifying the cell colonies, but a drawback of their approach is that they used a fixed window size of size 224 × 224 for localizing the cell colonies. This leads to smaller sized colonies to be overlooked leading to an incorrect segmentation.

**Fig 3:**
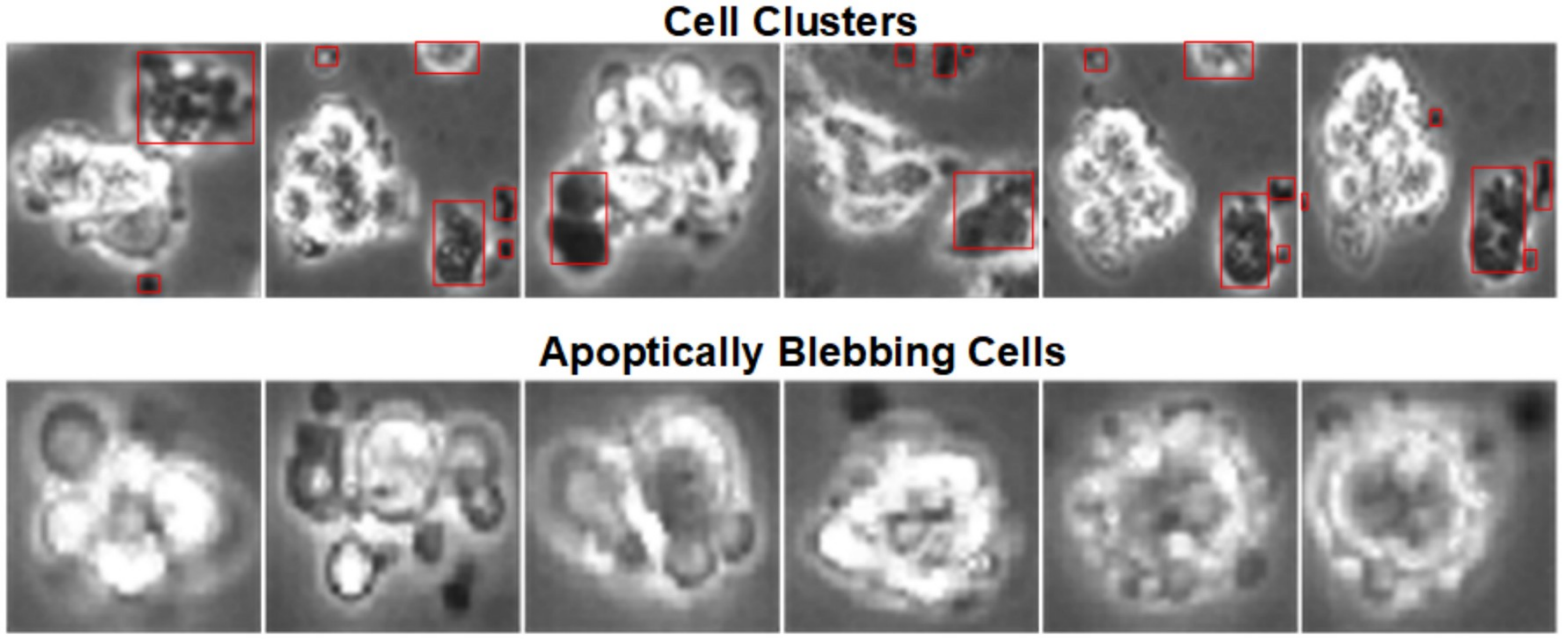
Example images of *Cell clusters* and *Apoptically Blebbing cells*. The distinguishing features between *Cell clusters* and *Apoptically Blebbing cells* are the small cells in the *Cell clusters* packed close to each other.

In our previous work using DeephESC [5], we used a CNN and Triplet CNNs to classify hESC images into six different classes. DeephESC was able to classify hESC images with an accuracy of 91.71%, but a problem encountered in this approach is that images belonging to the class *Cell clusters* were misclassifed as *Apoptically Blebbing cells* with an error rate of 7.89% which was the highest error percentage between any two classes. The reason for this is that *Cell clusters* and *Apoptically Blebbing cells* have a very similar texture and intensity. Fig 3 shows example images of *Cell clusters* and *Apoptically Blebbing cells*. The main distinguishing factor between these two classes is the presence of smaller cells packed close to the *Cell clusters*.

In Fig 3, the small cells in the manually annotated red bounding box are the factors that distinguish between a *Cell cluster* and an *Apoptically Blebbing cell*. Since these cells are very small, and as the image is passed forward through the convolution layers of CNN the dimensions of the feature maps progressively decrease and hence the receptive fields of the convolution filters are not able to detect these small cell bodies. To solve this DeephESC 2.0 skips connections between the initial and final convolution layers. The initial convolution layers learn a more coarse representation of the image where the receptive field of the filters are able to detect the small surrounding cells, whereas, the final layers learn a more fine-grained representation. By skipping intermediate layers and concatenating the feature maps of the initial and final convolution layers, DeephESC 2.0 is able to extract much more robust features that can detect these small surrounding cells which helps to improve the classification between these two classes.

### 2.3 Generation of synthetic hESC images

To the best of our knowledge, there is no published work that synthetically generates hESC images prior to our work in DeephESC [5]. Table 3 shows the summary of the related work for generating synthetic images of hESC. In DeephESC we evaluated two different approaches for generating synthetic hESC images namely: Deep Convolution Generative Adversarial Networks (DCGAN) [35] and ensemble—Deep Convolution Generative Adversarial Networks (e-DCGAN) [5].

**Table 3:** Summary of the related work for generating synthetic hESC images and contributions of this paper.

**Generation of synthetic hESC images**	Theagarajan *et al*. [5]	Used an ensemble of Deep Convolutional Generative Adversarial Networks (DCGAN) to generate synthetic images of six different classes of hESC. This approach pool together the features learned by individual DCGANs in order to improve the quality of the synthetic images.
	**Our Approach**	**This paper uses an ensemble of Generative Multi Adversarial Netwoks (GMAN) to generate synthetic hESC images for six different classes. By using multiple discriminators, the generator is able to learn a better feature representation of the original hESC images and hence generate higher quality synthetic images.**

Generative adversarial nets were recently introduced as a novel way to train a network to generate synthetic images. They consists of two ‘adversarial’ models: a generative model *G* that captures the data distribution, and a discriminative model *D* that estimates the probability that a sample came from the training data (real images) rather than the generator *G* (synthetic images). In order to learn the distribution *P_g_(x)* over data *x*, the generator builds a mapping function from a prior noise distribution *P_z_(z)* to data space as *G(z; θ_g_)*. The discriminator *D(x; θ_d_)* outputs a single scalar representing the probability that *x* came from the training data rather than *P_g_(x)*.

In DeephESC, we trained six different DCGANs (1 for each class) in order to generate synthetic hESC images. A problem encountered with this approach is that using DCGAN we were able to generate good quality images for all the classes except *Cell clusters*. The reason for this is that, *Cell clusters* have small cells packed very close to each other and the generator network for *Cell clusters* was not able to capture all the details in order to learn a good representation of the class. As a result the generated image had artifacts in it such as haloing and bleeding effects between cell boundaries.

**Fig 4:**
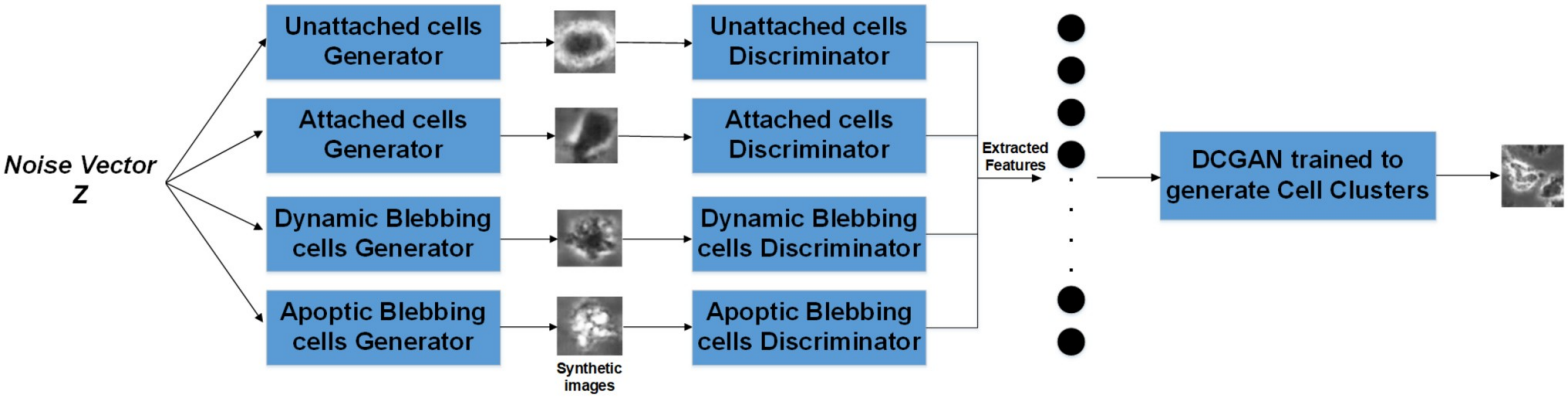
Archirecture of e-DCGAN for generating synthetic images of *Cell clusters*. A noise vector *Z* is given as input to the generators of the four intrinsic cells, the corresponding synthetic images are passed through their corresponding discriminators to extract a feature vector. The resulting feature vector is given as input to a DCGAN trained to generate synthetic images of *Cell clusters*.

To solve this we designed e-DCGAN an architecture that uses prior information from an ensemble of DCGANs to improve the quality of the generated images. By definition *Cell clusters* are a colony of two or more intrinsic cells (*Unattached*, *Attached*, *Dynamically Blebbing* and *Apoptically Blebbing cells*) packed close to each other. In order to generate synthetic images of *Cell clusters*, we used the features learned by the GANs corresponding to the four intrinsic cells in an ensemble manner as input to train another GAN to generate *Cell clusters*. Fig 4 shows the architecture of e-DCGAN.

In this paper, DeephESC 2.0 uses a variant of the DCGAN architecture named Generative Multi Adversarial Network (GMAN) [36]. GMAN is different from DCGAN by the fact that instead of using a single discriminator, we use *N* multiple discriminators to train the generator. In practice, training against a single discriminator can impede the generator’s learning. This is because if the generator is unlikely to generate any sample considered “realistic” by the discriminator’s standards, the generator will receive negative feedback. This is problematic because the information contained in the gradient derived from negative feedback only dictates where to drive down *P_g_(x)*, not specifically where to increase *P_g_(x)*. Furthermore, driving down *P_g_(x)* necessarily increases *P_g_(x)* in other regions of *X* (to maintain ∫_*X*_ *P_g_*(*x*) = 1) which may or may not contain samples from the true dataset (whack-a-mole dilemma). In contrast, a generator is more likely to see positive feedback against an ensemble of discriminators (because the generator needs to fool only 1 of the *N* discriminators), which may better guide a generator towards amassing *P_g_(x)* in approximately correct regions of *X*.

### 2.4 Contributions of this paper

To summarize, in comparison with the state-of-the-art and DeephESC, the contributions of DeephESC 2.0 are:

- An improved hierarchical classifier to classify hESC phase contrast image into six different classes with an accuracy of 93.23%
- Generating high quality synthetic hESC images using an ensemble of GMANs
- Exhaustive validation of the quality of the generated synthetic images using the Structural SIMilarity (SSIM) index, Peak Signal to Noise Ratio (PSNR) and statistical *p-* value tests.
- Training DeephESC 2.0 exclusively on a large amount of synthetic hESC images helps improve the classification accuracy of the classifier on the original hESC images from 93.23% to 94.46%.
- Comparison and visualization of the features learned using DeephESC [5] and DeephESC 2.0.

## 3 Materials and methods

### 3.1 Data

The hESC were cultured in vitro using methods described in detail previously [37]. The videos were acquired using the Nikon BioStation IM with a 20x objective resulting in a resolution of 600x800. A dataset of 784 cropped images was obtained from nine hESC videos. The dataset had the following numbers of images for each class: 1) 122 *Cell Cluster* images; 2) 113 *Debris* images; 3) 135 *Unattached cell* images; 4) 132 *Attached cell* images; 5) 104 *Dynamically Blebbing cell* images; and 6) 178 *Apoptotically Blebbing cell* images. The ground-truth for the dataset was annotated manually by expert stem cell biologists. The annotation was done by observing the morphology of the cells in the image as well as how they change in the video.

### 3.2 Methods for DeephESC 2.0

**Fig 5:**
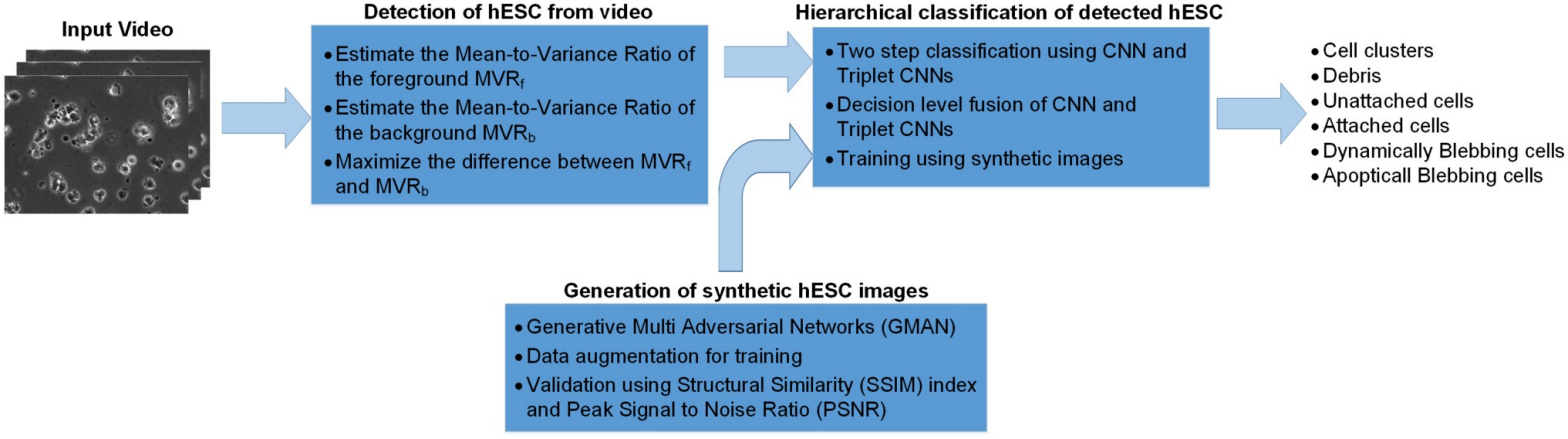
**Workflow of DeephESC 2.0 is split into three modules namely**: Detection of hESC from video, Generation of synthetic hESC images and hierarchical classification of the hESC images into six different classes.

DeephESC 2.0 is designed in a modular manner with three parts: hESC detection, hESC classification and hESC generation. Fig 5 shows the workflow of DeephESC 2.0. The source code was written and developed in PyTorch. The source code and supplied test data are available online at http://vislab.ucr.edu/SOFTWARE/software.php. To successfully run the source code requires the following softwares/libraries: python 3.5.2, pytorch 0.3.1, torchvision, PIL, numpy.

#### 3.2.1 Detection of hESC from videos using a mixture of Gaussians

**Fig 6:**
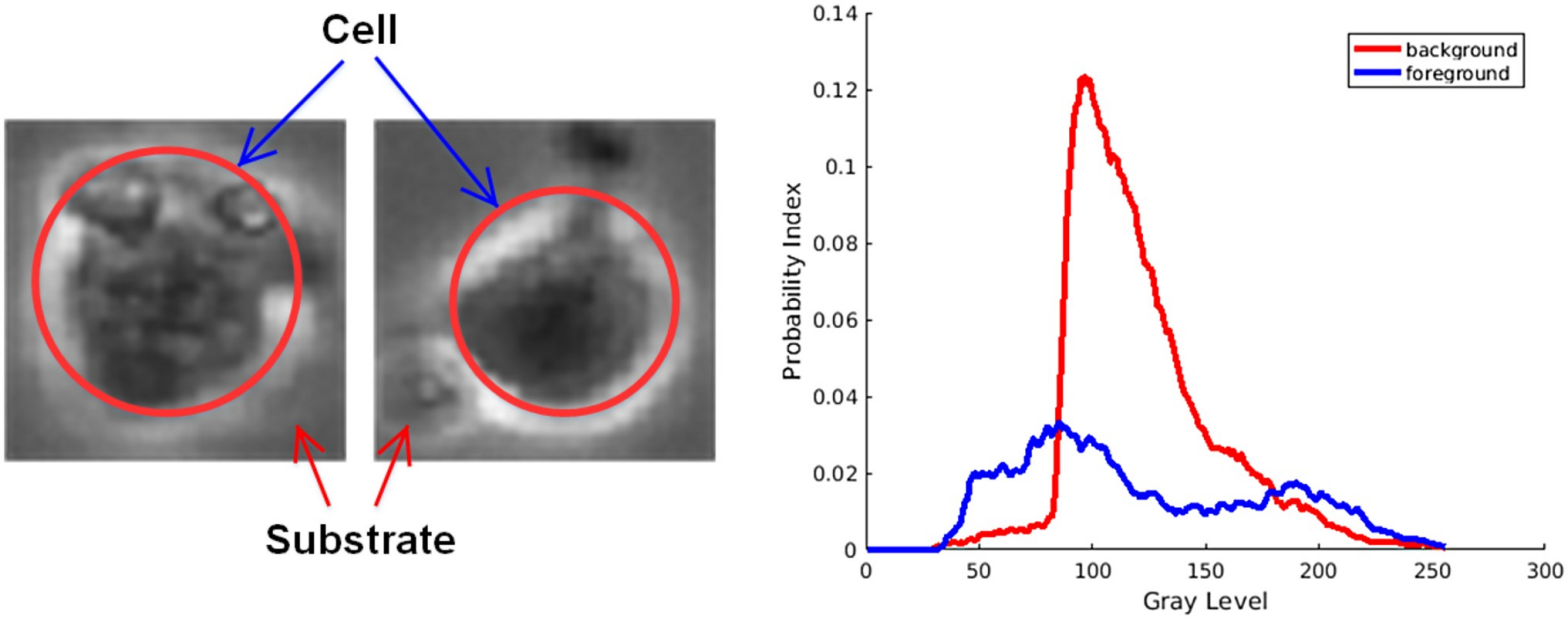
Images of the cell body and the substrate and their corresponding intensity distribution.

We detected and cropped stem cells from video frames of size 600 x 800 using a method developed by Guan *et al*. [24]. In the following we provide a brief description of the method. The hESC are grown in culture dishes coated with a layer of substrate (Matrigel). The substrate becomes the background after the hESC are placed on its surface. Therefore, we model a hESC image with two regions of interest: foreground and background [24]. Fig 6 shows examples of the cell (foreground) and the substrate (background) and their intensity distributions. Consequently we model the intensity distribution of foreground (cell region with a mean *μ_f_* and variance *σ_f_*^2^) and background (substrate region with a mean *μ_b_* and variance *σ_b_*^2^) as the mixture of two Gaussians.

With this model, we then want to maximize the absolute difference of mean-to-variance ratios of the foreground *MVR*_*f*_ and the mean-to-variance ratio of the background *MVR*_*b*_; The MVRs of the foreground and background data sets are calculated by the following equations:

$$\begin{array}{c} \begin{array}{c} MVR_{f}=\frac{\mu_{f}}{\sigma_{f}} \end{array} \end{array}$$ (1)

$$\begin{array}{c} \begin{array}{c} MVR_{b}=\frac{\mu_{b}}{\sigma_{b}} \end{array} \end{array}$$ (2)

where *MVR_f_* and *MVR_b_* are the MVRs for the foreground and background, respectively. Thus, the optimization metric *M* is formulated as:

$$\begin{array}{c} \begin{array}{c} M=|MVR_{f}-MVR_{b}| \end{array} \end{array}$$ (3)

substituting Eqs (1) and (2) into Eq (3), we get the following:

$$\begin{array}{c} \begin{array}{c} M=|\frac{\mu_{f}}{\sigma_{f}^{2}}-\frac{\mu_{b}}{\sigma_{b}^{2}}| \end{array} \end{array}$$ (4)

Eq (4) shows the metric that is used to determine how much the cell region data are different from the substrate region data. Since the algorithm is spatially evolving the foreground region from the initial high intensity variation region by a mean filter at each iteration, the foreground mean and variance are approaching to the background mean and variance. The limit of *M* is 0 as *μ*_*f*_/$\sigma_{f}^{2}$ approaches to *μ*_*b*_/$\sigma_{b}^{2}$. Therefore, our problem becomes finding *M_opt_* which is the optimal value for metric *M*, and the corresponding equation is described below:

$$\begin{array}{c} \begin{array}{c} M_{opt}=\underset{\mu_{\text{f}},\sigma_{\text{f}}{}^{2},\mu_{\text{b}},\sigma_{\text{b}}{}^{2}}{\text{max}}M\left(\mu_{\text{f}},\sigma_{\text{f}}{}^{2},\mu_{\text{b}},\sigma_{\text{b}}{}^{2}\right) \end{array} \end{array}$$ (5)

**Fig 7:**
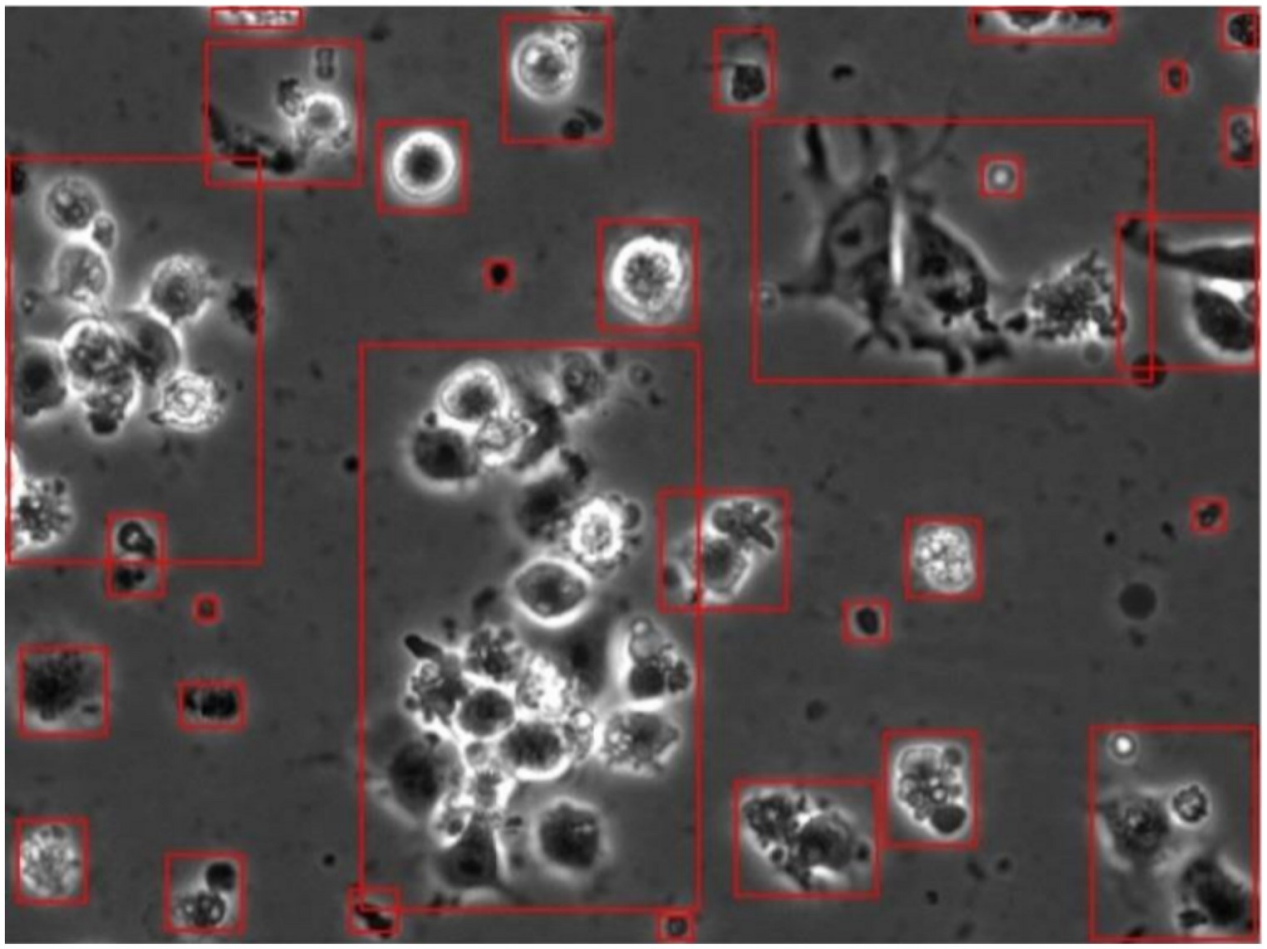
Detected cell bodies of a single frame using the approach proposed by [24]. The detected cell bodies are then cropped and passed through the hierarchical classifier to be classified into one of the aforementioned six classes.

*M_opt_* finds the parameters that maximize the difference between the foreground and background pixels. Fig 7 shows the detected components of a single frame. These detected components are then cropped and passed to the hierarchical classifier to be classified into one of the six aforementioned classes.

#### 3.2.2 Hierarchical classification of hESC

**Fig 8:**
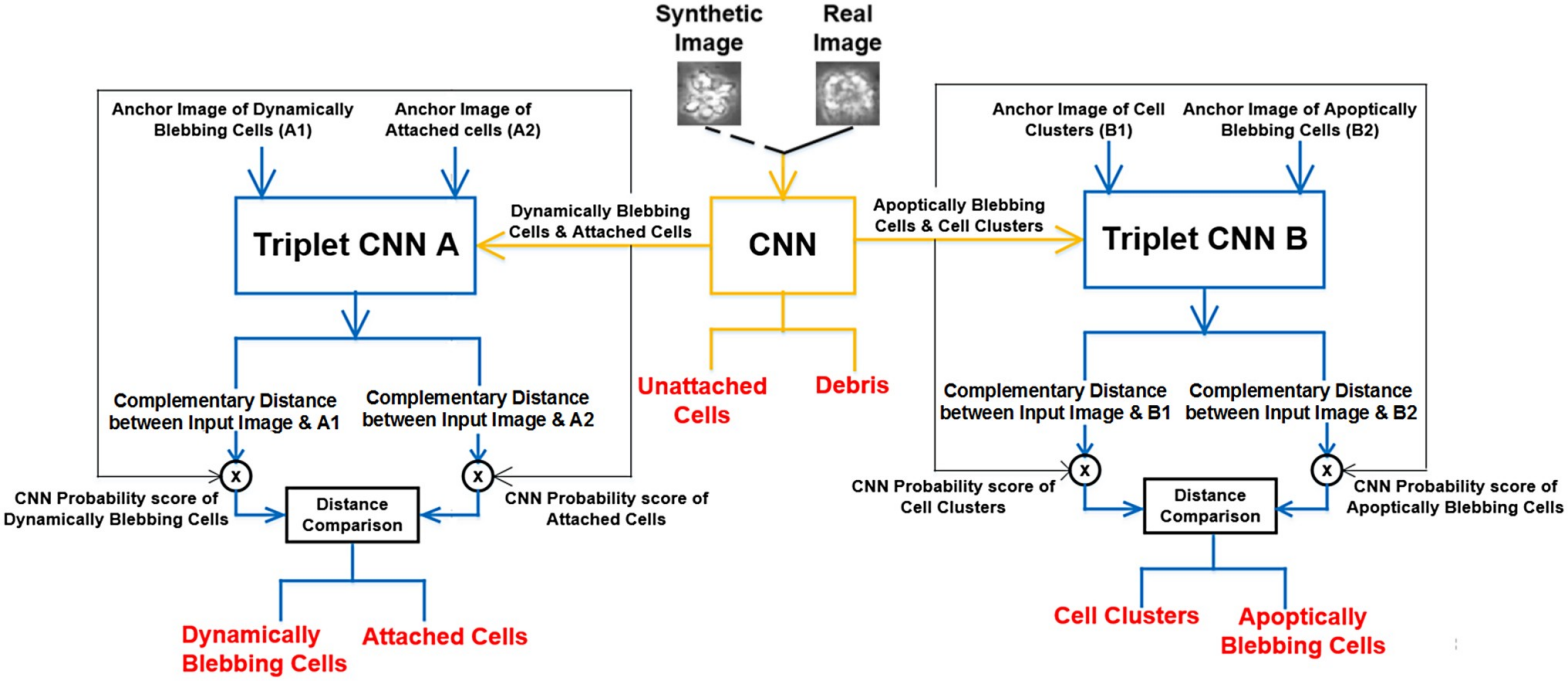
Workflow of the hierarchical classifier. The input is either a real or synthetic image belonging to one of the six classes. The outputs of the CNN and Triplet CNNs are fused at the decision level using the product rule.

In this section we explain in detail the architecture, training and parameters of the hierarchical classifier which includes the CNN and Triplet CNNs. Fig 8 shows the work flow of the hierarchical classifier.

**3.2.2.1 Convolution Neural Networks**: After detecting and cropping all the cell regions in a video, we resize all the hESC images to size 64x64. These images are then used for training the CNN. Table 4. shows the architecture details of our CNN. To train the CNN, we chose a mini batch size of 64. Since the size of our dataset is very limited, in order to prevent the CNN from over-fitting, we perform random affine transformations to the images and employ early stopping. Table 5 shows the data augmentation performed for training the CNN. We perform early stopping by saving the model after every epoch, only if the validation accuracy increases compared to the previous epoch. If the validation accuracy has not increased after 3 consecutive epochs we stop the training.

**Table 4:** Architecture of the Convolution Neural Network in the hierarchical classifier.

Input Dimension	Output Dimension	Number of feature maps	Layer (Kernel dimension, stride, padding)
64x64	32x32	64	Convolution (7, 2, 3)
32x32	16x16	64	Maxpool (3, 2, 1)
16x16	8x8	128	Convolution (5, 2, 2)
8x8	4x4	128	Maxpool (3, 2, 1)
2,048x1	6 classes	-	Fully connected layer

**Table 5:** Data augmentation performed to train the CNN.

Affine Transformation	Parameters
Image rotation	-180° to 180°
Image shearing	0° to 30°
Image zooming	70% to 140% of image size

We randomly chose 10 images from each class (60 images in total) as the validation dataset. The remaining of the dataset excluding the validation images, was divided into 5 folds for cross-validation. We did random hyper-parameter search for the CNN to obtain the best learning rate, momentum and weight decay. We chose random values for the learning rate, momentum and weight decay within a given range and step size and trained the network for three epochs. The combination of hyper-parameters that gave us the highest classification accuracy after three epochs are chosen as the best hyper-parameters for the network. The random hyper-parameter search was done by evaluating the CNN only on the validation dataset. Based on this we chose the best hyper-parameters as learning rate = 1.2x10^-2^, momentum = 0.9 and weight decay = 1x10^-3^ The network was optimized using the stochastic gradient descent algorithm with cross entropy loss.

We performed 5-fold cross validation and the results are shown in detail in the experimental section. After evaluating the CNN we observed that the CNN was able to classify the classes *Debris* and *Unattached Cells* with high accuracy, but the classes *Cell clusters*/*Apoptically Blebbing cells* and *Dynamically Blebbing Cells*/*Attached Cells* were misclassified the most. The reason for this is that, the classes *Cell clusters*/*Apoptically Blebbing Cells* and *Dynamically Blebbing Cells*/*Attached Cells* have similar intensity and texture. The only difference between these classes is their morphology.

**Fig 9:**
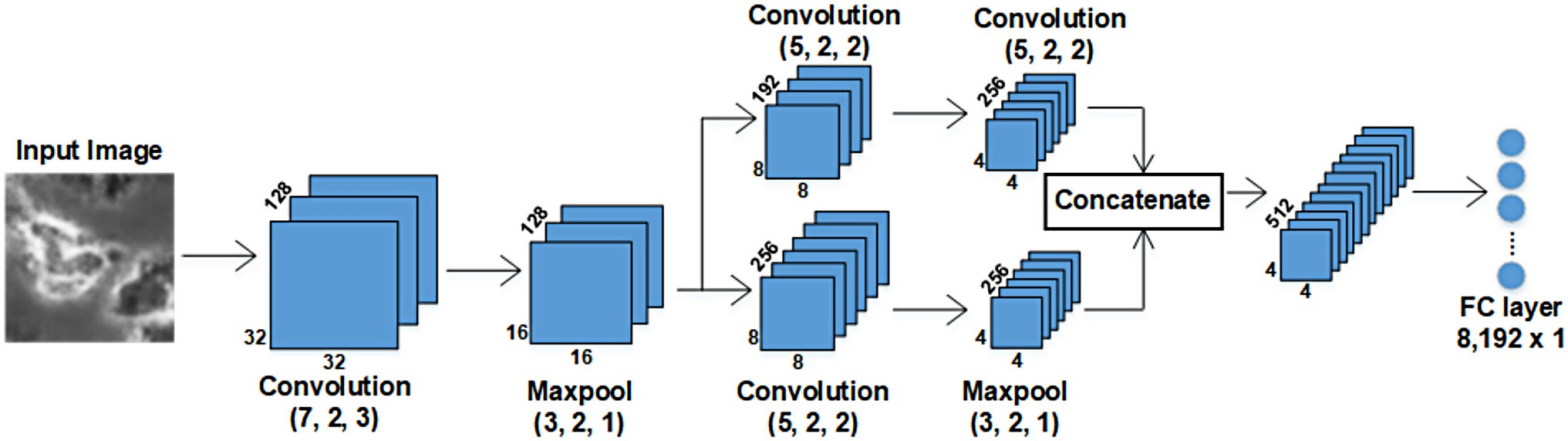
Architecture of Triplet CNN A and Triplet CNN B in Fig 8. The parameters within the parenthesis indicate the kernel dimension, stride and padding. By skipping intermediate layers and concatenating the feature maps of branched layers, DeephESC 2.0 is able to extract much more robust features, further improving the classification.

**3.2.2.2 Triplet Convolution Neural Network**: To solve this misclassification, we train a Triplet CNN to perform fine-grained classification between *Cell clusters* and *Apoptically Blebbing Cells* and similarly, for *Dynamically Blebbing Cells* and *Attached Cells*. Fig 9 shows the visual representation of the architecture for Triplet CNN A and Triplet CNN B from Fig 8.

The Triplet CNN architecture in Fig 9 is different from DeephESC by the fact that, DeephESC does not have any concatenation of feature maps between intermediate layers. By doing so, the initial convolution layers learn more coarse features while the final convolution layers are able to learn more fine-grained features. Concatenating the two branches together helps extract robust features and improves the classification accuracy compared to DeephESC [5]. The experimental results section shows the visual comparison of features extracted between DeephESC and DeephESC 2.0 and it can be observed that DeephESC fails to extract robust features for a given image compared to DeephESC 2.0.

The Triplet CNN takes as input a query image and one anchor image from each class. The output of the Triplet CNN is the two pairwise distances between the extracted features for the query image and the two anchor images as shown in Fig 8. For a correct classification, the pairwise distance between the query image and the anchor image belonging to the same class must be smaller (close to 0) compared to the distance between the query image and the anchor image belonging to the opposite class.

We used the same 10 validation images from each class used for validating the CNN, to validate the Triplet CNN. We randomly selected 5,000 triplet pairs for validation and 100,000 triplet pairs to train both Triplet CNN A and Triplet CNN B using 5-fold cross validation similar to how we trained the CNN. We chose a mini-batch size of 256 triplets and performed random hyper-parameter search and random affine transformation to the images as shown in Table 5 that was similarly done while training the CNN. Table 6. shows a summary for the best hyper-parameters for the CNN, Triplet CNN A and Triplet CNN B.

**Table 6:** Best hyper-parameters for training the networks in DeephESC 2.0.

Network	Learning rate	Momentum	Weight decay
CNN	1.2x10^-2^	0.9	1x10^-3^
Triplet CNN A	1.2x10^-2^	0.8	1x10^-3^
Triplet CNN B	2x10^-2^	0.8	1x10^-3^

The Triplet CNNs were optimized using the Stochastic Gradient Descent algorithm with the Ranked Marginal loss function given by Eq (6). In Eq (6), *X_1_* and *X_2_* are the two anchor images and *G(X)* is the pairwise distance between the feature extracted by Triplet CNN for the query image and the anchor image. In Eq (6) if *Y* = 1 it indicates that the anchor image *x_1_* belongs to the same class as the query image, whereas, *Y* = -1 indicates that the anchor image *x_2_* belongs to the same class as the query image. For all of our experiments we set the value of the margin as 1.

$$\begin{array}{c} \begin{array}{c} Loss=Max(0,-Y*(G(X_{1})-G(X_{2}))+margin) \end{array} \end{array}$$ (6)

Upon evaluating the Triplet CNNs with 5-folds cross validation, Triplet CNN A achieved an average classification accuracy of 95.24% and Triplet CNN B achieved an average classification accuracy of 95.83%.

**3.2.2.3 Decision level fusion of the CNN and Triplet CNNs**: After training the CNN and the individual Triplet CNNs we combine them in a hierarchical system as shown in Fig 8. The input hESC image is first passed into the CNN, the CNN is trained to classify the input image into one of the aforementioned six classes. If the predicted class is *Debris* or *Unattached cells*, we take the prediction of the CNN as the final prediction.

If the predicted class is *Attached cell* or *Dynamically Blebbing cells*, the input image is passed to Triplet CNN A, and we obtain the prediction of Triplet CNN A. Similarly, if the prediction of the CNN is *Cell cluster* or *Apoptically Blebbing cells*, the input image is passed to the Triplet CNN B and we obtain the prediction of Triplet CNN B.

The decision level fusion was done by taking the complementary pairwise distance (i.e. 1—pairwise distance) measure outputs from the Triplet CNN and multiplying the corresponding probability score for that class from the CNN. For example in Fig 8, in Triplet CNN B, the complementary pairwise distance measure between the input image and anchor image of *Cell clusters* is multiplied with the probability score for *Cell clusters* from the CNN. Similarly, the complementary pairwise distance measure between the input image and anchor image of *Apoptically Blebbing cells* is multiplied with the probability score for *Apoptically Blebbing cells* from the CNN, and so on for Triplet CNN A. The results obtained with and without the fusion are explained in detail in the experimental section.

#### 3.2.3 Generating synthetic hESC images using Generative Multi Adversarial Networks

The purpose of this section is to generate synthetic data and add more variability to the training dataset to help improve the classification performance of DeephESC 2.0. To achieve this we trained an ensemble of Generative Multi Adversarial Networks (GMAN) [36].

GMAN consists of a generator network *G* and *N* discriminator networks (*D_1_, D_2_, …, D_N_*). The generator takes a random noise vector *z* as input and returns an image *X*_*gen*_ = *G*(*z*). On the other hand, the discriminator takes a real or a generated image, and outputs a probability distribution *P*(*S*|*X*) = *D*(*X*) over the two image sources *S*. The discriminator is trained to maximize the log-likelihood of assigning the correct source while *G* tries to minimize it:

$$\begin{array}{c} \begin{array}{cc} \min_{G}\max_{D}V\left(D,G\right)= & E_{x∼p_{data}\left(x\right)}[\log D(x)]+\\ & +E_{x∼p_{z}\left(z\right)}[\log(1-D(G(z)))] \end{array} \end{array}$$ (7)

**Fig 10:**
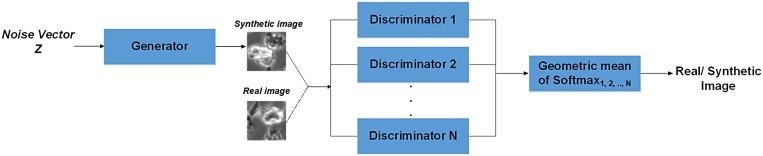
Architecture of GMAN. The generator is trained to take as input a random noise vector and generate an image that resembles the training data. The task of the *N* discriminators are to predict if the input image to the discriminator is either a real or a synthetic image. In our architecture of GMAN the softmax outputs of the *N* discriminators are combined together by computing their geometric mean.

In GMAN since we have multiple discriminators, we combine the outputs of the *N* discriminators using the weighted geometric mean as shown in Eq (8).

$$\begin{array}{c} \begin{array}{c} GM\left(V,\lambda \right)=-exp(\sum_{i}^{N}w_{i}log\left(-V_{i}\right)) \end{array} \end{array}$$ (8)

where, $w_{i}=e^{\lambda V_{i}}/\sum_{j}e^{\lambda V_{j}}$, *V*_*i*_ is the output of the *i*th discriminator and *λ* is a constant such that *λ* ≥ 0. The objective is that the generator network and the ensemble of discriminators converge to the Nash equilibrium so that *D*_1_, *D*_2_, …, *D*_*N*_ are maximally confused and *G* generates samples that resemble the training data. In our approach we trained six individual GMANs to generate images belonging to the corresponding six classes. Fig 10 shows the architecture of a GMAN. In DeephESC 2.0, we chose to use three different discriminators in our GMAN architecture. Table 7. shows the architecture of the generator and the three discriminators.

**Table 7:** Architecture of the generator and the three discriminators used in our Generative Multi Adversarial Network.

Network	Input dimension	Output dimension	Number of feature maps	Layer (Kernel dimension, stride, padding)
**Generator**	100x1	8,192x1	-	Fully connected layer
	4x4	8x8	256	ConvolutionT* (6, 2, 2)
	8x8	16x16	128	ConvolutionT* (6, 2, 2)
	16x16	32x32	64	ConvolutionT* (6, 2, 2)
	32x32	64x64	1	ConvolutionT* (6, 2, 2)
**Discriminator 1**	64x64	32x32	32	Convolution (5, 2, 2)
	32x32	16x16	64	Convolution (5, 2, 2)
	16x16	8x8	128	Convolution (5, 2, 2)
	8x8	4x4	256	Convolution (5, 2, 2)
	4,096x1	1	-	Fully connected layer
**Discriminator 2**	64x64	32x32	16	Convolution (5, 2, 2)
	32x32	16x16	32	Convolution (5, 2, 2)
	16x16	8x8	64	Convolution (5, 2, 2)
	8x8	4x4	128	Convolution(5, 2, 2)
	2,048x1	1	-	Fully connected layer
**Discriminator 3**	64x64	32x32	32	Convolution (5, 2, 2)
	32x32	16x16	64	Convolution (5, 2, 2)
	16x16	8x8	128	Convolution (5, 2, 2)
	8x8	4x4	256	Convolution (5, 2, 2)
	4x4	2x2	512	Convolution (5, 2, 2)
	2,048x1	1	-	Fully connected layer

* Note: ConvolutionT stands for the Convolution Transpose operation.

We chose the learning rate for the generator to be 1x10^-4^ and learning rate of the three discriminators to be 1x10^-5^ and mini batch of size 32. All the networks were optimized using the Adam algorithm [38] with loss function as a combination of Binary Cross Entropy and Embedding loss as shown in Eq (9).

$$\begin{array}{c} \begin{array}{c} Loss=\frac{-1}{n}\sum_{i=1}^{n}y_{i}*log\left(p_{i}\right)+\left(1-y_{i}\right)*log\left(1-p_{i}\right)\\ \;+\alpha *\frac{1}{n}\sum_{i=1}^{n}||X_{i}-X|{|}^{2} \end{array} \end{array}$$ (9)

In Eq (9), the first term is the Binary Cross Entropy loss. *y_i_* is the ground-truth label (real or synthetic image), *p_i_* is the probability score being a real image. The second term is the Embedding loss, *X_i_* is an image from the mini batch (either synthetic or real image) and *X* is a real image chosen randomly from the training dataset belonging to the same class as *X_i_*. The Binary Cross Entropy loss ensures that the GMAN is able to extract accurate features to generate synthetic images resembling the images from the training dataset and the Embedding loss ensures that the generated images have a similar morphology as the images from the training dataset. *α* is an empirical value and was chosen to be 5x10^-2^.

## 4 Experimental results

### 4.1 Detection of hESC from video

We evaluated the detection of hESC objects using the algorithm proposed by Guan *et al*. [24]. The metrics used for evaluating the detection are Jaccard similarity, Dice coefficient, Specificity and Sensitivity. The Sensitivity (SEN), measures the proportion of actual positives which are correctly detected:

$$\begin{array}{c} \begin{array}{c} SEN=\frac{TP}{\left(TP+FN\right)} \end{array} \end{array}$$ (10)

The Specificity (SPC), is the true negative rate which is given by:

$$\begin{array}{c} \begin{array}{c} SPC=\frac{TN}{\left(FP+TN\right)} \end{array} \end{array}$$ (11)

The Jaccard similarity (J), is a measure of similarity between the detected results and the ground-truth:

$$\begin{array}{c} \begin{array}{c} J=\frac{TP}{\left(TP+FP+FN\right)} \end{array} \end{array}$$ (12)

The Dice coefficient (DIC), measures the agreement between the detected results and the ground-truth:

$$\begin{array}{c} \begin{array}{c} DIC=\frac{2TP}{\left(2TP+FP+FN\right)} \end{array} \end{array}$$ (13)

The approach achieved a Jaccard similarity (J) of **0.754**, Dice coefficient (DIC) of **0.860**, Sensitivity (SEN) of **0.906** and Specificity (SPC) of **0.924**.

### 4.2 Measures for classification performance

We trained and evaluated the classifier using the *K*- fold cross validation. *K*- fold cross validation divides the dataset into *K* subsets. Each time, one of the *K* subsets is used as the testing set and the remaining *K*—1 subsets are put together to form a training set. Then the average error across all *K* trials is computed. The advantage of this method is that it matters less how the data gets divided. Every data point gets to be in the testing set exactly once, and gets to be in a training set *K*—1 times. The variance of the resulting estimate is reduced as *K* is increased. In the following we evaluated the classification accuracy using the 5- fold cross validation (*K* = 5).

#### 4.2.1 Classification results

Table 8 shows the average classification accuracy for the 5-fold cross validation using CNN, CNN-Triplet and Fused CNN-Triplet approach of DeephESC 2.0 and Tables 9, 10 and 11 show the confusion matrices for the CNN, CNN-Triplet and fused CNN-Triplet, respectively. All the networks in Table 8 were trained and evaluated on the real hESC images. We compare the results obtained using DeephESC 2.0 with the results obtained using DeephESC. The dataset has a total of 784 real hESC images, 10 randomly chosen images from each class (60 in total) were used as the validation dataset. In order to maintain fairness in evaluation, these 60 validation images were not used for evaluating the performance of the networks. The remaining 724 hESC images are split into 5 folds for cross validation. Note that the results shown in Tables 8–11 are for the 724 images used in the 5 fold cross validation.

**Table 8:** Comparison of the average classification accuracy of the networks used in DeephESC and DeephESC 2.0.

Approach	Network	Average Classification Accuracy
**ResNet18** [39]	CNN	70.44%
**VGG19** [40]	CNN	72.57%
**AlexNet** [14]	CNN	71.91%
**DeephESC** [5]	CNN	86.14%
	CNN-Triplet	89.37%
	Fused CNN-Triplet	91.71%
**DeephESC 2.0**	CNN	**86.33**% ± 0.29
	CNN-Triplet	**90.88%** ± 0.26
	Fused CNN-Triplet	**93.23%** ± 0.24

**Table 9:** Confusion matrix for the classification of the 724 real hESC images using the CNN architecture of DeephESC 2.0.

**Class**	**CC**	**DEB**	**UN**	**AT**	**DYN**	**APO**
**CC**	**97**	3	0	0	1	11
**DEB**	0	**100**	1	1	1	0
**UN**	2	0	**121**	1	0	1
**AT**	1	2	0	**100**	16	3
**DYN**	2	0	1	10	**81**	0
**APO**	30	4	2	1	5	**126**

**Table 10:** Confusion matrix for the classification of the 724 real hESC images using the CNN-Triplet architecture of DeephESC 2.0.

**Class**	**CC**	**DEB**	**UN**	**AT**	**DYN**	**APO**
**CC**	**102**	3	0	0	1	6
**DEB**	0	**100**	1	1	1	0
**UN**	2	0	**121**	1	0	1
**AT**	1	2	0	**105**	11	3
**DYN**	2	0	1	4	**87**	0
**APO**	13	4	2	1	5	**143**

**Table 11:** Confusion matrix for the classification of the 724 real hESC images using the Fused CNN-Triplet architecture of DeephESC 2.0.

**Class**	**CC**	**DEB**	**UN**	**AT**	**DYN**	**APO**
**CC**	**105**	3	0	0	1	3
**DEB**	0	**100**	1	1	1	0
**UN**	2	0	**121**	1	0	1
**AT**	1	2	0	**110**	6	3
**DYN**	2	0	1	2	**89**	0
**APO**	6	4	2	1	5	**150**

The Abbreviations used in Tables 9, 10 and 11 are as follows: **CC**: *Cell clusters*, **DEB**: *Debris*, **UN**: *Unattached cells*, **AT**: *Attached cells*, **DYN**: *Dynamically Blebbing cells*, **APO**: *Apoptically Blebbing cells*.

Comparing Tables 9 and 10 it can be observed that, the misclassification between the classes ***Cell clusters*** **(CC)** and ***Apoptically Blebbing cells*** **(APO)** has been reduced from 14.64% to 6.79% using the CNN-Triplet compared to just the CNN. Similarly, the misclassification of ***Attached cells*** **(AT)** and ***Dynamically Blebbing cells*** **(DYN)** has been reduced from 12.04% to 6.94%. Moreover, upon fusing the outputs of the CNN and the Triplet CNN we further reduced the misclassification of ***Cell clusters*** **(CC)** and ***Apoptically Blebbing cells*** **(APO)** to **3.21%** and the the misclassification of ***Attached cells*** **(AT)** and ***Dynamically Blebbing cells*** **(DYN)** to **3.70%**.

#### 4.2.2 Comparison of features learned by DeephESC 2.0 and DeephESC

**Fig 11:**
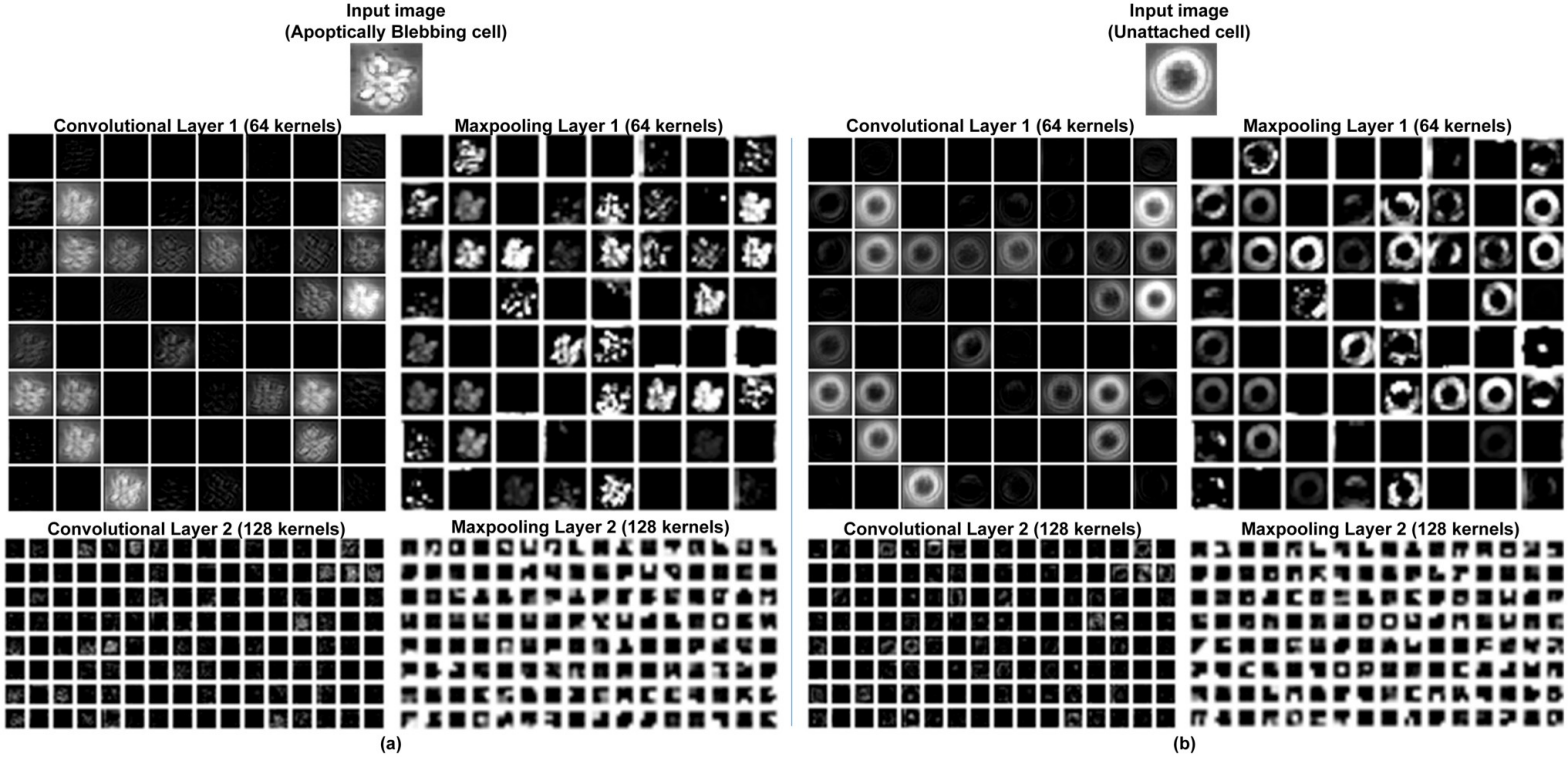
Visualization of features extracted by the CNN in DeephESC 2.0 for (a) *Apoptically Blebbing cell* and (b) *Unattached cell*.

Fig 11(a) and 11(b) shows the features extracted by the CNN used in DeephESC 2.0. for an *Apoptically Blebbing cell* and *Unattached cell* respectively. In Fig 11, the first convolutional layer learns filters some of which look like edge detectors, filters for image blurring and image sharpening. These features become more sparse and localized as the data flows further through the layers of the CNN.

**Fig 12:**
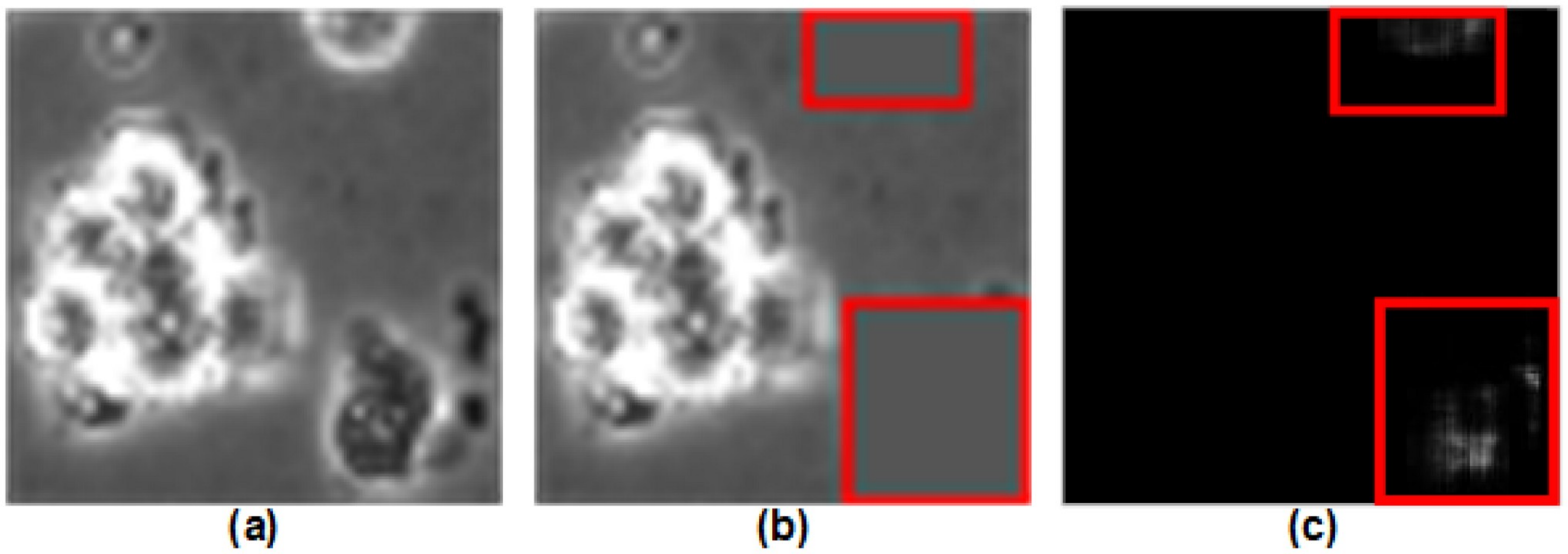
Visualization of features learned by DeephESC 2.0. (a) Image of a *Cell cluster*. (b) Image after masking the surrounding small cells using a window. Red bounding boxes are drawn across the masked area only for visualization purposes. (c) Probability heat map for the class *Apoptically Blebbing cell*.

In order to compare the improvement in classification between DeephESC and DeephESC 2.0, we visualized the features learned by DeephESC 2.0. Fig 12(a) shows an image of a *Cell cluster* (CC) that was correctly classified by DeephESC 2.0, but was incorrectly classified as *Apoptically Blebbing cell* (APO) by DeephESC.

We masked the area containing the surrounding small cells in Fig 12(a) with a sliding window of size 5 x 5 with gray scale pixel value of 85 (pixel range is from 0 to 255) that matches the surrounding background as shown in Fig 12(b). For visualization purposes we draw a red bounding box across the masked area in Fig 12(b). The image in Fig 12(b) is then passed through the hierarchical classifier for each position of the sliding window and the output probability score of the class *Apoptically Blebbing cell* (APO) for that center position of the sliding window is plotted in Fig 12(c).

The inference that we get from Fig 12(c) is that, the bright pixel locations indicate the locations that the classifier predicts as important features for the image being a *Cell cluster*. The reason for this is that, the 5 x 5 mask window centered around that area is masking the small cells as seen in Fig 12(b), and since the network is unable to see these surrounding small cells, it predicts the image to be an *Apoptically Blebbing cell*. Hence, this means that the small cells in the image are considered as important features for the network to classify the image as a *Cell Cluster*.

### 4.3 Synthetic hESC images from GMAN

**Fig 13:**
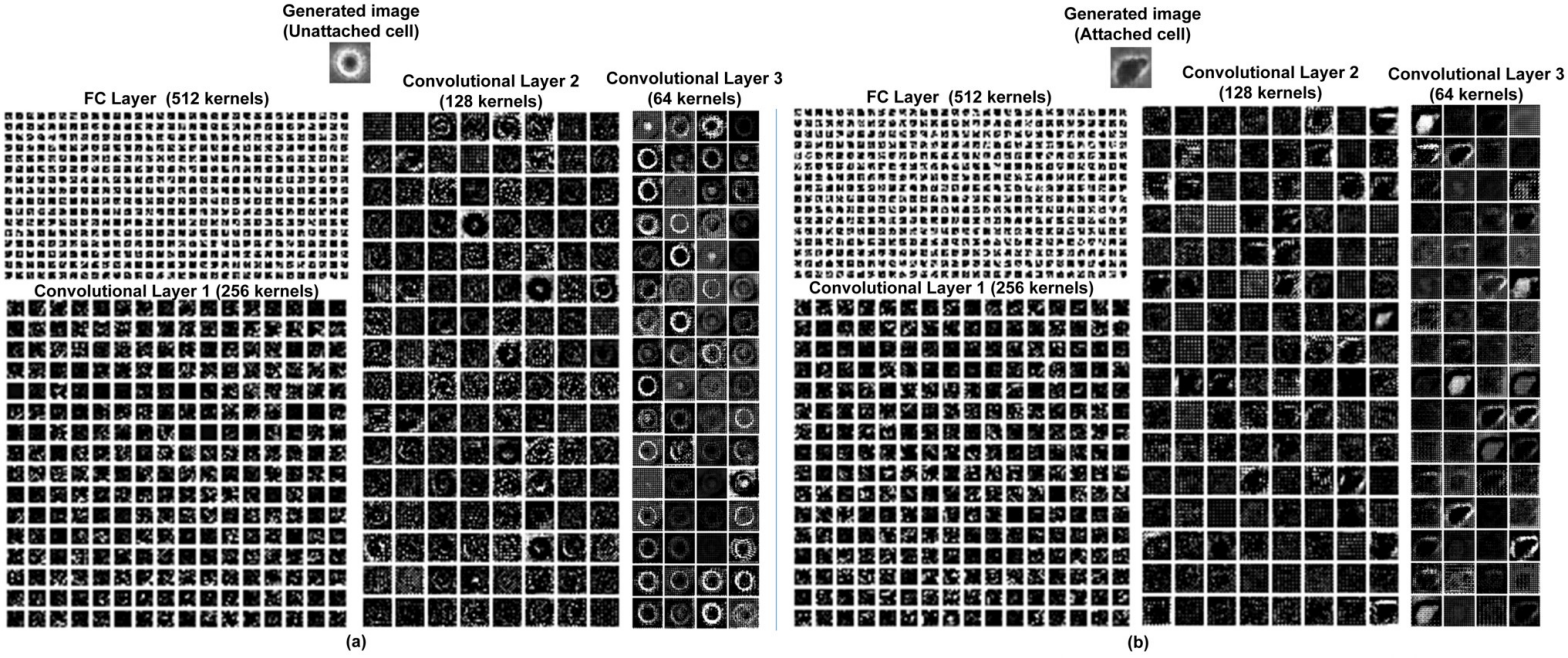
Visualization of features learned by the generators in DeephESC 2.0. (a) *Unattached cell* and (b) *Attached cell*.

Fig 13(a) and 13(b) shows examples for visualizing the features learned by the generators in DeephESC 2.0 for generating an *Unattached cell* and *Attached cell*, respectively. In Fig 13, the input to the respective generators is a 100x1 dimensional randomly sampled Gaussian noise vector. We can observe that the FC layer and the first convolutional layer learn features that are very sparse and localized. As these features progress through the layers of the generator, the features become more smooth and gradually start to resemble a hESC both in texture and shape.

#### 4.3.1 Evaluation of the quality of the generated synthetic images

**Fig 14:**
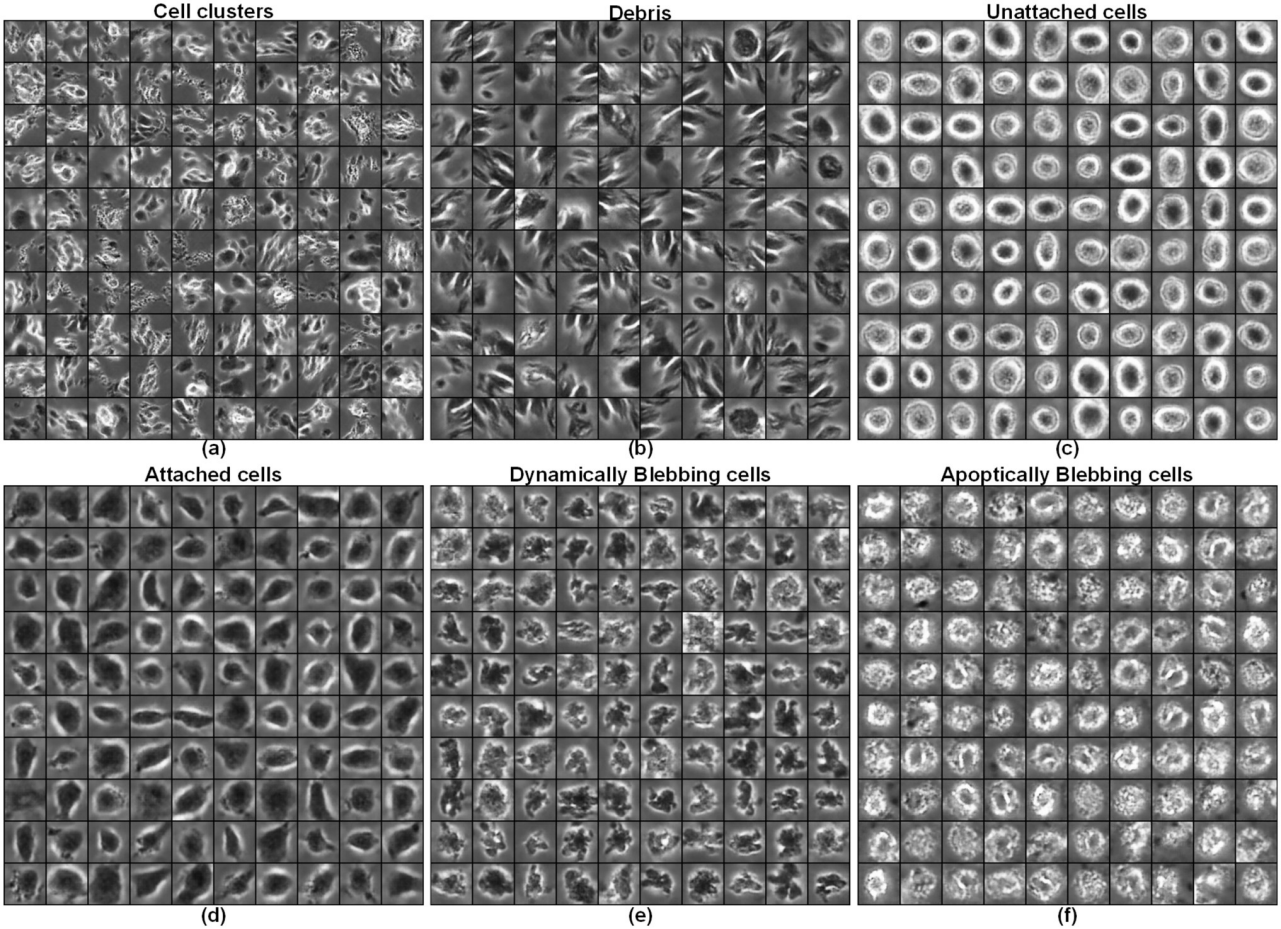
The 600 synthetic images used for validating the quality in Table 12. (a) *Cell clusters*, (b) *Debris*, (c) *Unattached cells*, (d) *Attached cells*, (e) *Dynamically Blebbing cells*, (f) *Apoptically Blebbing cells*.

In order to evaluate the quality of the synthetic images, we first generated 100 synthetic images for each of the six classes. Fig 14 shows the 600 synthetic images that were generated for validating the quality. The average Structural Similarity (SSIM) score and average Peak-Signal-to-Noise Ratio (PSNR) score for a given synthetic image are computed by computing average the SSIM and PSNR between that given synthetic image and all the real images in the dataset for that given class. This is repeated for all the 100 synthetic images in each class and the average SSIM score and PSNR score is obtained. The structural similarity index between two images is calculated by:

$$\begin{array}{c} \begin{array}{c} SSIM\left(X,Y\right)=\frac{\left(2\mu_{x}\mu_{y}+C_{1}\right)\left(2\sigma_{xy}+C_{2}\right)}{\left(\mu_{x}^{2}+\mu_{y}^{2}+C_{1}\right)\left(\sigma_{x}^{2}+\sigma_{y}^{2}+C_{2}\right)} \end{array} \end{array}$$ (14)

In Eq (14), *μ*_*x*_ and *μ*_*y*_ are the average pixel values of image *X* and *Y* respectively, $\sigma_{x}^{2}$ and $\sigma_{y}^{2}$ are the variance of the pixel values of image *X* and *Y*, respectively, *σ*_*xy*_ is the covariance between image *X* and *Y*. *C*_1_ and *C*_2_ are constants given by *C*_1_ = (*K*_1_ *L*)^2^ and *C*_2_ = (*K*_2_ *L*)^2^, where, *L* = 255 is the maximum range of the pixel values and *K*_1_ = 0.01 and *K*_2_ = 0.03 are fixed constants. The PSNR between two images is calculated by:

$$\begin{array}{c} \begin{array}{c} PSNR\left(X,Y\right)=10\;log_{10}(\frac{L^{2}}{MSE(X,Y)}) \end{array} \end{array}$$ (15)

In Eq (15), *L* = 255 is the maximum range of the pixel values, *MSE*(*X*, *Y*) is computed by $MSE\left(X,Y\right)=\frac{1}{mn}\sum_{i=1}^{m}\sum_{j=1}^{n}{\left[X\left(i,j\right)-Y\left(i,j\right)\right]}^{2}$, *m* and *n* are the spatial dimensions of the synthetic image *X* and real image *Y*. Table 12 shows the average SSIM score and PSNR score obtained using the 100 synthetic images for each class shown in Fig 14.

**Table 12:** Comparison of our GMAN architecture used in DeephESC 2.0 with e-DCGAN [5], DCGAN [35] and c-DCGAN [41] using the SSIM and PSNR metrics. SSIM has no units and PSNR is measured in decibels (dB).

Approach	Metric	Cell Cluster	Debris	Unattached cell	Attached cell	Dynamically Blebbing cell	Apoptically Blebbing cell
**GMAN**	Avg. SSIM	**0.6312**	**0.6217**	**0.8347**	**0.6072**	**0.5921**	**0.5827**
	Avg. PSNR	**19.71**	**18.23**	**26.27**	**17.56**	**16.25**	**16.82**
**e-DCGAN** [5]	Avg. SSIM	0.6047	0.5931	0.7731	0.5730	0.5463	0.5498
	Avg. PSNR	18.23	15.77	24.29	16.28	14.33	14.24
**DCGAN** [35]	Avg. SSIM	0.5732	0.5931	0.7731	0.5730	0.5463	0.5498
	Avg. PSNR	18.23	15.77	24.29	16.28	14.33	14.24
**c-DCGAN** [41]	Avg. SSIM	0.5691	0.5722	0.7231	0.5897	0.5625	0.5411
	Avg. PSNR	18.27	15.28	20.96	15.29	15.33	15.09

The scale for SSIM is from 0—1 and has no unit, 0 indicates the images have no resemblance and 1 indicates they are the same images. The ideal range for SSIM score is from 0.5—0.85. The scale for PSNR is from 0—∞ and is measured in dB, 0 indicates the images have no similarity and ∞ indicates they are the same images. The ideal range for PSNR score is from 15dB—30dB.

From Table 12, it can be observed that our GMAN architecture achieved the highest average SSIM and PSNR score for all the six classes. *Unattached cells* had the highest SSIM and PSNR score of 0.8347 and 26.27 dB, respectively as this class of hESC was the easiest to generate. The reason for this is that *Unattached cells* visually have the least complex structure compared to the other five classes. This is further supported by the observation that *Unattached cells* had a high correct classification accuracy of 96.80% because they are very easy to classify. It should also be noted that, the SSIM and PSNR for e-DCGAN and DCGAN are the same except for the class *Cell clusters* because both of these approaches use the same architecture of generators and discriminators for all the classes except *Cell clusters*.

### 4.4 Augmenting the dataset

Since SSIM and PSNR metrics tend to ignore the higher order characteristics of the image, we evaluated the quality of the synthetic images by training the classifier using different proportions of real and synthetic images. The assumption of this approach is that, if the synthetic images have similar higher order characteristics compared to the real images, then the features learned by the CNNs during the training on the synthetic images, should also be able to classify the real images.

To verify this assumption, we trained and evaluated our hierarchical classifier in two different data settings:

- Training on 100% real images.
- Training on 100% synthetic images.

Training on 100% real images is the same experiment as reported in Table 8. Table 13 shows the accuracy for each fold in the 5-fold cross validation using the 724 real hESC images. In the second data setting, we trained our fused CNN-Triplet classifier exclusively on the synthetic images and evaluate the performance on the real hESC images. Table 14 shows the accuracy after training the classifier using different amounts of synthetic images.

**Table 13:** Accuracy and number of images of each fold for the 5-fold cross validation using the 724 real hESC images. The number in the brackets indicates the number of images per class for Cell clusters, Debris, Unattached cells, Attached cells, Dynamically blebbing cells, and Apoptically blebbing cells respectively.

Cross validation fold number	Number of images for training	Number of images for testing	Classification accuracy
**Fold 1**	580 (88, 80, 100, 100, 76, 136)	144 (24, 23, 25, 22, 18, 32)	93.18%
**Fold 2**	579 (90, 83, 100, 97, 75, 134)	145 (22, 20, 25, 25, 19, 34)	93.02%
**Fold 3**	579 (90, 83, 100, 97, 75, 134)	145 (22, 20, 25, 25, 19, 34)	93.65%
**Fold 4**	579 (90, 83, 100, 97, 75, 134)	145 (22. 20, 25, 25, 19, 34)	93.21%
**Fold 5**	579 (90, 83, 100, 97, 75, 134)	145 (22, 20, 25, 25, 19, 34)	93.10%
**Average**	-	-	**93.23 ± 0.24%**

**Table 14:** Comparison of using different data compositions of synthetic images for training the classifier and then testing it on the 724 real images.

Number of synthetic hESC images per class used for training	Classification Accuracy on the 724 real hESC images
**5,000**	93.84%
**10,000**	94.26%
**20,000**	94.31%
**30,000**	94.43%
**40,000**	**94.46%**

Observing the results in Table 14, it can be seen that training the classifier exclusively with the synthetic images resulted in an increase in the classification accuracy. This verifies our assumption that the generated synthetic images do have similar higher order characteristics as the real images and hence augmenting our dataset helps the classifier to generalize better resulting in an increase in classification accuracy.

We verified the significance of the accuracy in Table 14 using the statistical *p*-value test. The *p*-value is calculated using the one-way Analysis of Variance (one-way ANOVA). One-way ANOVA is a technique that can be used to compare means of two or more experiments using the F distribution. We assume the training using real images in Table 13 and the training using synthetic images in Table 14 to be two different experiments. Based on this setting, the one-way ANOVA yields a F score ratio of 33.18, which corresponds to a *p*-value of 4.24 × 10^−4^. We set the significance threshold of the *p*-value as 0.01. Since, the *p*-value (4.24 × 10^−4^) is lower than the threshold (0.01), our results are proved to be significant.

### 4.5 Discussion of results

In this section we discuss about the improvement in classification accuracy, quality of the generated synthetic images and the reasons for misclassification.

#### 4.5.1 Improvement in classification accuracy

This subsection explains the reasons for the improvement in classification accuracy compared to our prior work in DeephESC [5]. We show that by concatenating feature maps from the early and final stages of the CNN, the CNN learns a better feature representation and helps reduce the misclassification between visually similar classes.

It can be observed from Table 11 that *Debris* and *Unattached cells* had the highest classification accuracy of 97.08% and 96.80%, respectively. The reason for this is that these two classes are visually very distinctive compared to *Cell clusters*/*Apoptically Blebbing cells* and *Attached cells*/*Dynamically Blebbing cells*.

On the contrary, in comparison with our prior work in DeephESC [5]*Cell clusters*/ *Apoptically Blebbing cells* had the highest misclassification rate of 7.89%. The reason for this is that the CNN was not able to detect the small neighboring cells which distinguish a *Cell cluster* from an *Apoptically Blebbing cell* as depicted in Fig 3. In DeephESC 2.0 we solved this by concatenating features learned from the initial and final convolution layers which helps the CNN learn a more robust feature representation as shown in Fig 12 which in turn reduces the misclassification rate from 7.89% to 3.21%. Similarly *Attached cells*/*Dynamically Blebbing cells* have very similar intensities and texture with the only difference being in their morphology. *Attached cells* have a more uniform and homogeneous morphology compared to *Dynamically Blebbing cells*. By concatenating the features from initial and final convolution layers we are able to reduce the misclassification rate from 5.26% to 3.70%.

#### 4.5.2 Quality of the generated synthetic images

This subsection explains why Unattached cells have higher SSIM and PSNR scores compared to the other five classes. We also explain the disadvantage of using SSIM and PSNR to validate the quality of the images and how we overcome this problem.

It is observed from Table 12 that the SSIM and PSNR for the five classes *Cell clusters*, *Debris*, *Attached cells*, *Dynamically* and *Apoptically Blebbing cells* were relatively lower compared to the SSIM and PSNR for Unattached cells. The reason for this is that the structure of these five classes are much more complex and diverse compared to Unattched cells as shown in Fig 14. SSIM and PSNR metrics compare the similarity between two images at a pixel level ignoring the higher order characteristics (such as the overall structure and texture). Although our approach is able to generate synthetic images which visually look similar to the original images, due to the diverse variations in shape even a slight change in corresponding pixel values will result in a significantly low SSIM and PSNR value.

Since SSIM and PSNR tends to ignore higher order characteristics of the image, we evaluated the quality of higher order characteristics of the synthetic images by training our classifier exclusively on the synthetic image and tested its classification accuracy on the real hESC images as shown in Table 14. The assumption here is that, if the real hESC images and the generated synthetic images have similar higher order characteristics, then the features learned by the CNN trained on the synthetic images should be able to also classify the real hESC images. From Table 14, we can observe that our CNN trained exclusively on synthetic images is able to classify the real hESC images with an accuracy of 94.46%. This observation validates our assumption that the generated synthetic images do have similar higher order characteristics as the real hESC images.

#### 4.5.3 Saturation of classification accuracy

This subsection shows how the accuracy of the classifier varies with increasing amounts of synthetic images as well as the trade-off between the number of images for training Vs the time taken for training. We also show some examples of hESC images that were predicted incorrectly by our classifier and explain the reason for the misclassification.

**Fig 15:**
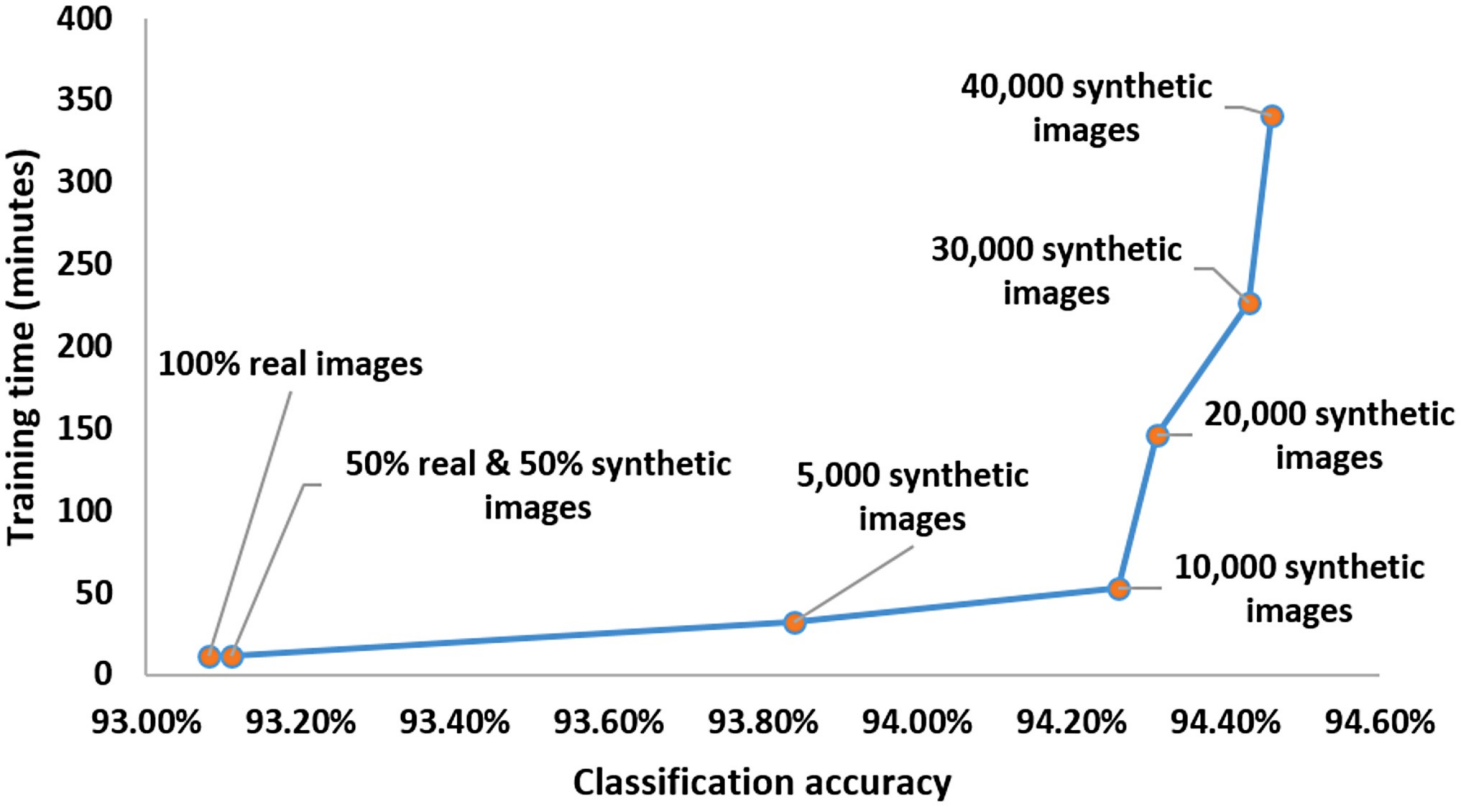
Classification accuracy Vs training time trade-off.

It can be observed from Table 14, the classification accuracy increases progressively as we generate more synthetic images, but after a certain amount of synthetic images (40,000 synthetic images per class) the classification accuracy does not significantly increase. In Table 14 we get an improvement in accuracy of only 0.03% from increasing the number of synthetic images from 30,000 to 40,000 per class but the time taken to train the classifier significantly increases. Hence, in order to balance the trade-off between the classification accuracy and the training time we limit the number of synthetic images per class to be 40,000. Fig 15 shows the graph of the classification accuracy versus the training time trade-off.

**Fig 16:**
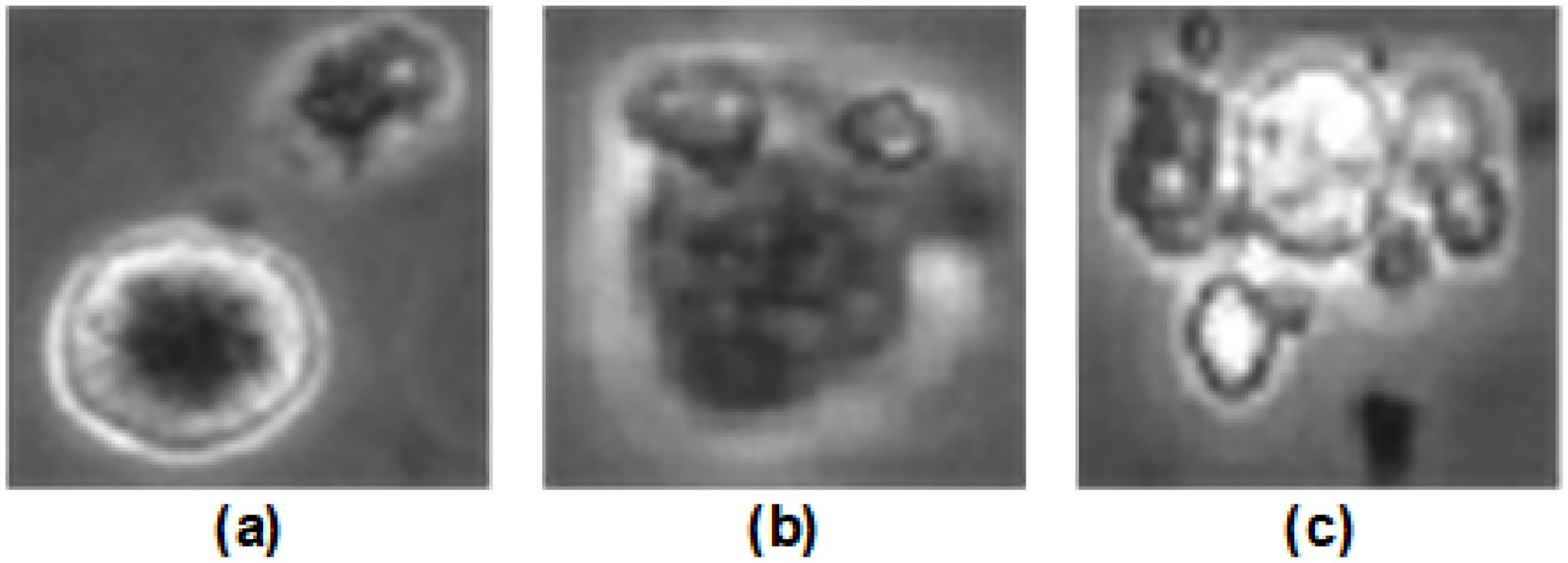
Examples of images that were unintentionally labeled wrong by the biologist, but correctly classified by our classifier. (a) *Unattached cell* mislabeled as *Cell cluster*, (b) *Attached cell* mislabeled as *Dynamically Blebbing cell*. (c) *Apoptically Blebbing cell* mislabeled as *Cell cluster*.

A possible reason for the saturation in classification accuracy is that the ground-truth for certain images may have been labeled incorrectly by the biologists and the classifier is able to correctly classify these images even though the ground-truth is wrong. Fig 16 shows examples of such images that were unintentionally labeled incorrectly by the biologist, but our classifier was still able to predict the correct class.

Fig 16(a) is an *Unattached cell*, but due to the presence of a growing *Dynamic Blebbing cell* near it, the biologist decided to label it as a *Cell cluster*. Fig 16(b) is a *Dynamically Blebbing cell* that was mislabeled as an *Attached cell*. Since the morphology of these two classes are very similar, the biologist was not sure to which class the hESC belonged to. Fig 16(c) is a *Cell cluster* mislabeled as *Apoptically Blebbing cell*. This is another example where the morphology of of two classes look very similar and the biologist was not sure as to which class the hESC belonged to.

## 5 Conclusions

We proposed DeephESC 2.0 an automated system for detecting and classifying hESC images. DeephESC 2.0 outperforms our prior work done in DeephESC [5] in both the classification and generation of synthetic hESC images. We observed that the certain classes such as *Cell clusters*/*Apoptically Blebbing cells* and *Attached cells*/*Dynamically Blebbing cells* have similar texture and intensity and they are only different in their morphology. To exploit this difference we designed Triplet CNN architectures with branched convolution layers that can detect these minute changes in morphology and perform fine-grained classification for further improving the classification accuracy of these classes. Moreover, by fusing the outputs of the CNN and Triplet CNNs using the product rule we were able to further improve the classification accuracy to 93.23%. We also showed the difference between DeephESC 2.0 and DeephESC in terms of the learned features, and observed that DeephESC 2.0 was able to learn more robust features that could detect the presence of small *Cell clusters* where DeephESC failed.

We designed individual GMANs for each class to generate synthetic hESC images. We evaluated the quality of the generated images using the SSIM, PSNR and statistical *p-* value metrics and our approach outperformed state-of-the-art approaches for generating synthetic hESC images. Furthermore, we trained the classifier of DeephESC 2.0 exclusively on 40,000 synthetic images per class and evaluated the classifier on the real hESC images and achieved further improved classification accuracy of 94.46%. We discussed the possible reasons for misclassification and observed that some images were unintentionally mislabeled by the biologists and our approach was able to predict their correct class. This shows that our approach is robust even in the presence of noisy data.
